# Loss of CMD2‐mediated resistance to cassava mosaic disease in plants regenerated through somatic embryogenesis

**DOI:** 10.1111/mpp.12353

**Published:** 2016-04-05

**Authors:** Getu Beyene, Raj Deepika Chauhan, Henry Wagaba, Theodore Moll, Titus Alicai, Douglas Miano, James C. Carrington, Nigel J. Taylor

**Affiliations:** ^1^ Donald Danforth Plant Science Center 975 North Warson Road St. Louis MO 63132 USA; ^2^ National Crops Resources Research Institute Namulonge P. O. Box 7084 Kampala Uganda; ^3^ University of Nairobi, P. O. Box 29053 Nairobi post code 00625 Kenya

**Keywords:** cassava, cassava mosaic disease, CMD2, geminivirus resistance, somaclonal variation, somatic embryogenesis, susceptible

## Abstract

Cassava mosaic disease (CMD) and cassava brown streak disease (CBSD) are the two most important viral diseases affecting cassava production in Africa. Three sources of resistance are employed to combat CMD: polygenic recessive resistance, termed CMD1, the dominant monogenic type, named CMD2, and the recently characterized CMD3. The farmer‐preferred cultivar TME 204 carries inherent resistance to CMD mediated by CMD2, but is highly susceptible to CBSD. Selected plants of TME 204 produced for RNA interference (RNAi)‐mediated resistance to CBSD were regenerated via somatic embryogenesis and tested in confined field trials in East Africa. Although micropropagated, wild‐type TME 204 plants exhibited the expected levels of resistance, all plants regenerated via somatic embryogenesis were found to be highly susceptible to CMD. Glasshouse studies using infectious clones of *East African cassava mosaic virus* conclusively demonstrated that the process of somatic embryogenesis used to regenerate cassava caused the resulting plants to become susceptible to CMD. This phenomenon could be replicated in the two additional CMD2‐type varieties TME 3 and TME 7, but the CMD1‐type cultivar TMS 30572 and the CMD3‐type cultivar TMS 98/0505 maintained resistance to CMD after passage through somatic embryogenesis. Data are presented to define the specific tissue culture step at which the loss of CMD resistance occurs and to show that the loss of CMD2‐mediated resistance is maintained across vegetative generations. These findings reveal new aspects of the widely used technique of somatic embryogenesis, and the stability of field‐level resistance in CMD2‐type cultivars presently grown by farmers in East Africa, where CMD pressure is high.

## Introduction

Cassava mosaic geminiviruses (CMGs) are the causal pathogens of cassava mosaic disease (CMD) which affects cassava production in tropical Africa and the Indian subcontinent. CMGs are single‐stranded bipartite DNA viruses belonging to the family *Geminiviridae*, genus *Begomoviru*s, and are transmitted by the whitefly *Bemisia tabaci*. The viruses are further disseminated by the planting of infected stem cuttings used by farmers to establish the next cropping cycle (Hillocks and Thresh, 2000; Legg, 1999). Diseased plants show mosaic symptoms and distortion of the leaves, leading to significant suppression of storage root yields (Legg *et al*., 2006). CMD was recently listed within the top 10 most important viruses affecting crop plants (Scholthof *et al*., 2011).

Three sources of host plant resistance are known and currently exploited by breeders to combat CMD. The first was introgressed from *Manihot glaziovii* (ceara rubber) during early breeding programmes in Africa (Nichols, 1947). This mechanism was later found to be polygenic and recessive in nature (Hahn *et al*., 1980), with the gene(s) conferring this resistance mapped and named as CMD1 (Fregene and Puonti‐Kaerlas, 2002). Cultivars possessing CMD1‐type resistance have been widely deployed in East and West Africa, mostly as the Tropical Manihot Species (TMS) series (Okogbenin *et al*., 2013) developed by breeding programmes at the International Institute for Tropical Agriculture (IITA). A second source of CMD resistance was discovered within related landraces collected from farmers' fields in Nigeria and other West African countries during the 1980s and 1990s (IITA, 1990; Okogbenin *et al*., 2013). Resistance in these landraces, which carry the prefix TME (Tropical *Manihot esculenta*), originates from a monogenic, dominant locus, named CMD2 (Akano *et al*., 2002). Landraces possessing CMD2‐mediated resistance were imported to East Africa and widely deployed and adopted by farmers in response to the CMD epidemic that occurred in Uganda in the 1990s (Legg *et al*., 2006). The development of molecular markers (Akano *et al*., 2002) and the ease of introgression of this single dominant locus has made the CMD2‐type germplasm highly favoured by breeders in Africa as they strive to develop improved varieties (Rabbi *et al*., 2014). The third resistance mechanism exists as a quantitative trait locus (QTL), named CMD3 (Okogbenin *et al*., 2012), identified in cultivar TMS 97/2205. Although CMD3 confers very high levels of resistance to CMD with little or no expression of disease on the leaves (Okogbenin *et al*., 2012), both CMD1‐ and CMD2‐type plants become infected with CMGs and develop typical mosaic symptoms. Subsequently, however, a recovery phenotype is observed by which the severity of CMD symptoms displayed on new growth reduces over time, until the newly formed leaves are free of visible disease (Okogbenin *et al*., 2013). The gene(s) involved and the molecular mechanisms underlying CMD1, CMD2 and CMD3 resistance remain unknown.

Unlike CMD, effective resistance to cassava brown streak disease (CBSD) has not been found within existing cassava germplasm, although cultivars displaying varying levels of tolerance have been reported (Abaca *et al*., 2012; Kaweesi *et al*., 2014; Patil et al., 2015). CBSD is caused by *Cassava brown streak virus* (CBSV) and *Ugandan cassava brown streak virus* (UCBSV) (Monger *et al*., 2001; Winter *et al*., 2010), both of which are positive‐sense, single‐stranded RNA viruses belonging to the family *Potyviridae*, genus *Ipomovirus*. CBSD poses a present and imminent threat to cassava production in East and Central Africa through yield suppression and the development of necrotic lesions within the storage roots of infected plants (Legg *et al*., 2011). Efforts have been undertaken to generate CBSD resistance in cassava germplasm using RNA interference (RNAi) technologies (Ogwok *et al*., 2012; Taylor *et al*., 2012; Vanderschuren *et al*., 2012). Transgenic plant lines have been generated in the farmer‐preferred cultivar TME 204 (Chauhan *et al*., 2015), employing a construct designed to generate small interfering RNAs (siRNAs) against the coat proteins (CPs) of both CBSV and UCBSV. TME 204 was originally collected from Benin (Lokko *et al*., 2006) and was considered to carry innate CMD2‐mediated resistance to CMD. We report the finding that all regenerated RNAi TME 204 plants lose their inherent resistance to CMD. Evidence is provided showing that this phenomenon is not related to the production of siRNAs against CBSD or the genetic transformation process *per se*, but results from the passage of tissues through somatic embryogenesis. A similar loss of CMD resistance occurs in the CMD2‐type cultivars TME 7 and TME 3, but was not observed in CMD1‐ and CMD3‐type cultivars.

## Results

### Loss of resistance to CMD in cultivar TME 204 under field and glasshouse conditions

**Figure 1:**
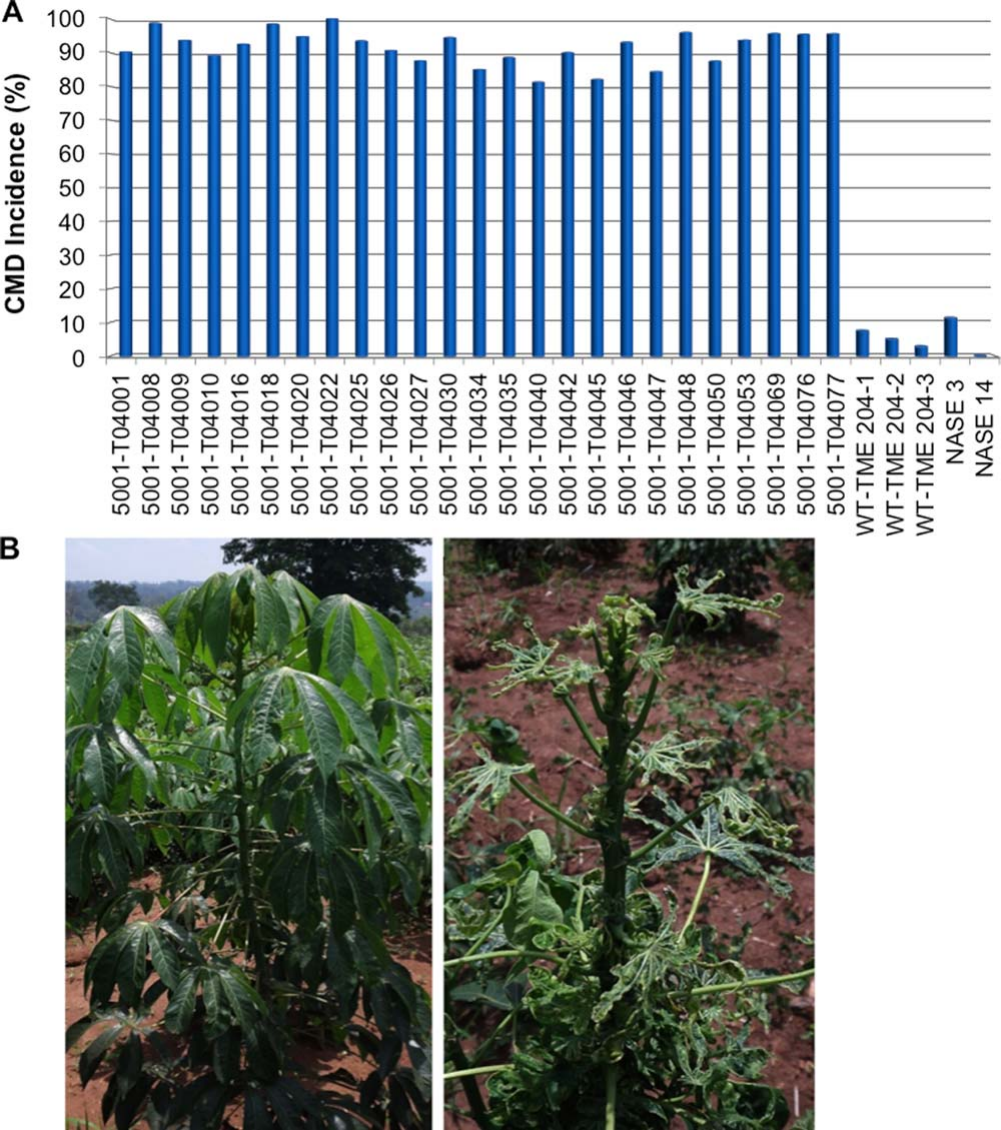
Response of RNA interference (RNAi) p5001 and wild‐type TME 204 lines to cassava mosaic disease (CMD) under confined field trial conditions at the National Crops Resources Research Institute (NaCRRI), Namulonge, Uganda. p5001 lines express an inverted repeat of coat protein genes derived from *Ugandan cassava brown streak virus* and *Cassava brown streak virus* fused in tandem. (A) Incidence of CMD in p5001 TME 204 plant lines at 6 months after planting. Data collected from eight replicates (10 plants each) per line. (B) Wild‐type (left) and p5001 CMD symptomatic (right) plants.

The CMD‐resistant cultivar TME 204 was modified with the RNAi construct p5001 in an effort to integrate resistance to CBSD. This RNAi construct carries an inverted repeat of CP genes derived from UBCSV and CBSV fused in tandem. *Agrobacterium*‐mediated transformation of friable embryogenic callus (FEC) of TME 204 was used to generate transgenic p5001 lines (Chauhan *et al*., 2015). Molecular analysis of the 450 independent lines produced identified a subset possessing one to two copies of the T‐DNA and expressing siRNAs from the CP sequences of CBSV and UCBSV (data not shown). Twenty‐five such independent lines were micropropagated, transferred to Namulonge, Uganda and established under confined field trial (CFT) conditions in a manner similar to that previously described by Ogwok *et al*. (2012). Non‐transgenic plants of TME 204 micropropagated from tissue culture stocks were also established in the field to serve as wild‐type controls. CMD symptoms were first observed on all TME 204 plants at 1–2 months after planting (MAP). The incidence and severity of disease continued to increase on RNAi plants, but not on the wild‐type controls. The latter were observed to undergo the typical recovery phenotype, producing symptom‐free new growth and recovering to show little or no visible CMD on the young leaves. By 6 MAP, all 25 RNAi lines displayed CMD at incidences of 80%–100% (Fig. 1A), increasing to 100% of the plants (*c*. 2000 in total) by 12 MAP (Fig. S1, see Supporting Information). In contrast, the micropropagated non‐transgenic plants displayed CMD at an average of approximately 5.3% and 10.3% at 6 and 12 MAP, respectively (Figs 1A and S1).

Experiments were carried out under controlled growth conditions at the Donald Danforth Plant Science Center (DDPSC), St. Louis, MO, USA to investigate the unexpected loss of resistance to CMD observed in the field. The goal was to determine whether the loss of resistance to CMD resulted from unknown field‐specific conditions, whether transgenic production of siRNAs within the modified TME 204 was responsible or whether the causal factors resided within the genetic transformation and tissue culture system used to produce these plants. Additional CMD2‐, CMD1‐ and CMD3‐type cultivars were also studied to determine whether the loss of resistance observed was specific to cultivar TME 204 alone.

**Figure 2:**
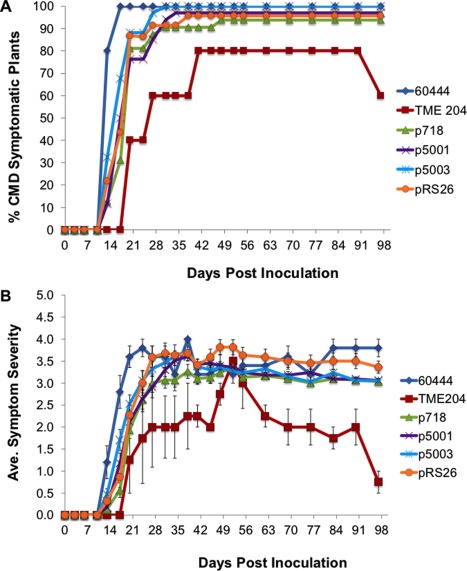
Response of transgenic TME 204 lines to biolistic inoculation with an infectious clone of *East African cassava mosaic virus* (EACMV‐K201) under glasshouse conditions. Percentage of cassava mosaic disease (CMD) symptomatic plants (A) and average symptom severity scores (scale 0–5) (B) of transgenic lines expressing an inverted repeat of coat protein genes derived from *Ugandan cassava brown streak virus* (UCBSV‐CP) and *Cassava brown streak virus* (CBSV‐CP) fused in tandem (p5001, six lines), transgenic lines expressing the inverted repeat of CBSV‐CP (p5003, four lines) or UCBSV‐CP (p718, four lines), transgenic lines expressing the iron assimilatory protein gene *FEA1* (pRS26, three lines) and a susceptible control cv. 60444. Bars show standard error (*n* = 4–34).

TME 204 plant lines transgenic for p5001 (Chauhan *et al*., 2015), p5003 (inverted repeat derived from the CP gene of CBSV) and p718 (inverted repeat derived from the CP gene of UCBSV (Fig. S2, see Supporting Information), all designed to produce CP‐derived siRNAs against CBSD, were established in the glasshouse. Plants were inoculated with the infectious clone EACMV‐K201 by Helios® microparticle delivery into young leaves. All transgenic lines of p5001 (six independent lines), p5003 (four independent lines) and p718 (four independent lines) and the susceptible control 60444 became infected with EACMV‐K201 (83%–100%) with average symptom severities of 3.0–3.5 (0–5 scale) (Figs 2A,B and S3, see Supporting Information). The maximum number of plants showing CMD symptoms and the maximum symptom severity scores were attained 3–5 weeks after virus inoculation. Transgenic plants and the susceptible cultivar 60444 did not recover from CMD and retained severe symptoms (scores 3.0–4.0) for the remaining 11‐week observation period (Figs 2A,B and S3). In addition, 80% of non‐transgenic wild‐type TME 204 plants that had been clonally propagated from tissue culture stocks developed CMD symptoms with an average severity score of 3.5. However, these plants then recovered from the disease, with CMD severity reducing over time to reach an average score of 1.2 by 11 weeks after inoculation (Figs 2A,B and S3).

### Loss of CMD resistance is not caused by the presence of transgenes or *Agrobacterium*‐mediated transformation

Two experiments were undertaken to determine whether acquired susceptibility to CMD was related to the production of transgene‐derived siRNAs or a result of the genetic transformation process. For the former, transgenic p5001 TME 204 plants were inoculated with EACMV‐K201, together with TME 204 plants transgenic for pRS26 which harbours the *FEA1* transgene (see Fig. S2), non‐transgenic wild‐type TME 204 and non‐transgenic plants of the known CMD‐susceptible cultivar 60444. Expression of the iron assimilatory protein (*FEA1*) transgene was designed for iron biofortification of cassava storage roots (Ihemere *et al*., 2012) and these plants do not produce siRNAs against the virus. The development of CMD indicated that the pRS26 transgenic plant lines were equally as susceptible to challenge with EACMV‐K201 as the p5001 siRNA‐producing lines (Figs 2A,B and S3). In both cases, the transgenic plants did not undergo the recovery phenotype associated with wild‐type TME 204 plants. Indeed, CMD symptom development and severity scores observed in the pRS26 and p5001 transgenic lines were comparable with those displayed by the highly CMD‐susceptible cv. 60444 (Figs 2A,B and S3).

**Figure 3:**
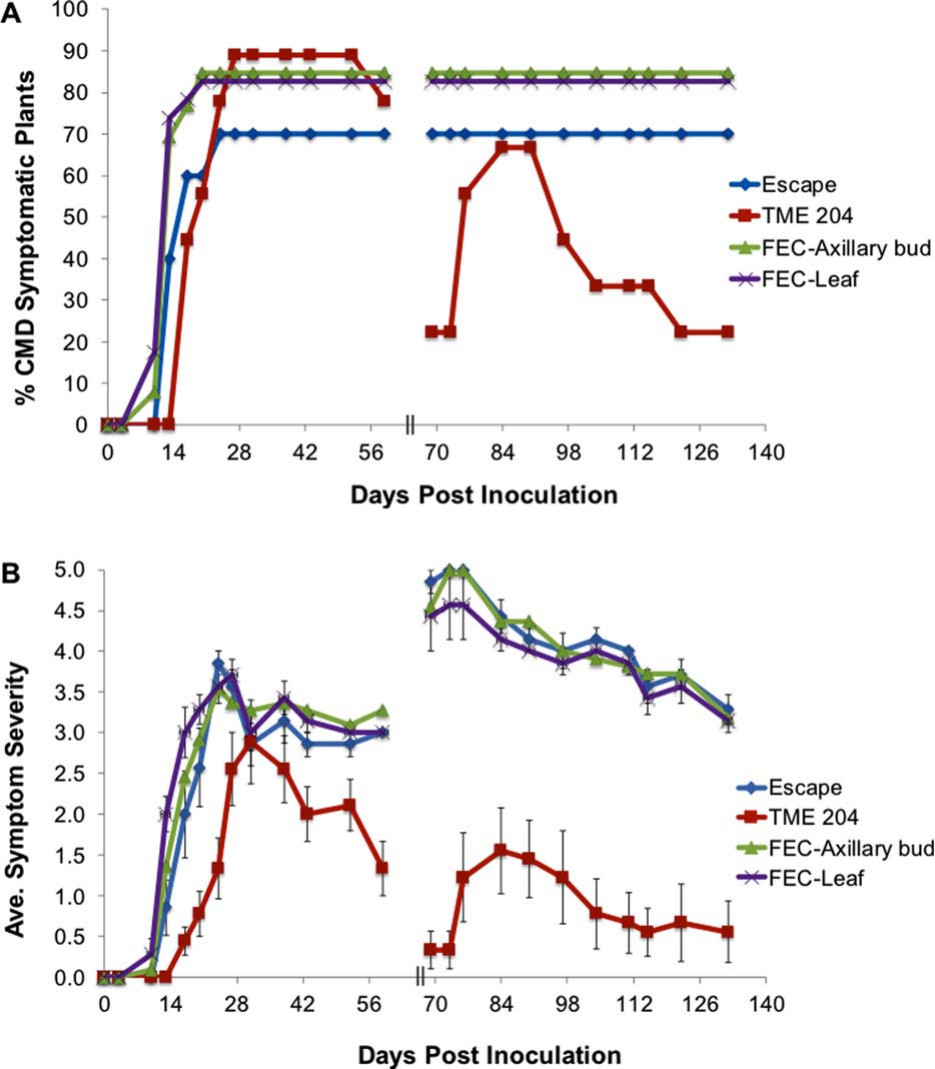
Response of friable embryogenic callus (FEC)‐derived plants of TME 204 to inoculation with an infectious clone of *East African cassava mosaic virus* (EACMV‐K201) under glasshouse conditions. (A) Percentage of cassava mosaic disease (CMD) symptomatic plants. (B) Average symptom severity scores (scale 0–5). Cassava plantlets were recovered from FEC derived from axillary bud (FEC‐Axillary bud) and leaf (FEC‐Leaf) explants without exposure to *Agrobacterium* and subsequent antibiotic selection. Escapes were plants recovered from the transformation process including exposure to *Agrobacterium* and subsequent antibiotic selection processes, but confirmed to be lacking the T‐DNA. Plant stems were cut back at 60 days after biolistic inoculation and CMD was assessed on new leaf growth. Breaks in the *x* axis indicate a lapse in shoot regrowth after this cut back. Bars show standard error (*n* = 7–11).

Having established that the loss of resistance to CMD was not caused by the presence of siRNAs targeted against CBSD, investigations were undertaken to assess the effects of the tissue culture processes used to generate the plants (Chauhan *et al*., 2015; Taylor *et al*., 2012). During the production of the p5001‐expressing plants, some lines of TME 204 were regenerated, but subsequently found to be negative for the presence of the T‐DNA. These plant lines, termed ‘escapes’, were exposed to all stages of embryogenic tissue production, *Agrobacterium* inoculation and antibiotic selection. Escape plants were inoculated with EACMV‐K201 in parallel with plants regenerated from FEC derived from both leaf and axillary buds (Bull *et al*., 2009; Taylor *et al*., 2012) that had not been exposed to the transformation system. Escape and FEC‐derived plants developed CMD symptoms (70%–81%) within 3–4 weeks after inoculation and with minimal recovery from the disease over the subsequent 4 weeks (Fig. 3A,B). In contrast, the wild‐type control plants showed progressive recovery from a maximum severity of 3.0 observed at 4.5 weeks to an average score of 1.3 at 8.5 weeks post‐inoculation.

To study the recovery phenotype in more detail, the stems of all plants were cut back to 12.5 cm above soil level at 8 weeks post‐inoculation. New shoot growth showed mild CMD symptoms with an average severity score of 1.6 on TME 204 wild‐type plants, followed by subsequent recovery from disease, such that the number of plants showing CMD symptoms reached less than 25% with average severity scores of 0.6 by 14 weeks after inoculation (Fig. 3A,B). New leaves of both escape and FEC‐derived plants, from both explant sources, developed very severe CMD, retaining an average score of 3.1 and above until the end of the observation period (Fig. 3A,B). Failure to recover from CMD in FEC‐derived and escape plants indicated that the acquired susceptibility to CMGs seen in the transgenic lines was not caused by T‐DNA integration, the type of explant used to induce FEC or specific factors, such as the antibiotics used during the transformation and selection process (Chauhan *et al*., 2015). Instead, elevated susceptibility and failure to recover from infection with cassava geminivirus was associated with the passage of tissues through the disorganized FEC tissue (Fig. 3A,B).

### Loss of CMD2‐type resistance is not unique to TME 204

**Figure 4:**
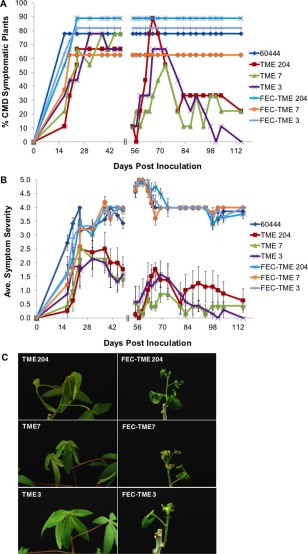
Response of friable embryogenic callus (FEC)‐derived and micropropagated wild‐type plants of CMD2‐type cultivars to inoculation with an infectious clone of *East African cassava mosaic virus* (EACMV‐K201) under glasshouse conditions. FECs were generated and plants recovered from TME 3, TME 7 and TME 204. (A) Percentage of cassava mosaic disease (CMD) symptomatic plants. (B) Average symptom severity scores (scale 0–5). Plant stems were cut back at 48 days after biolistic inoculation and CMD was assessed on new leaf growth. Breaks in the *x* axis indicate a lapse in shoot regrowth after this cut back. Bars show standard error (*n* = 5–9). (C) CMD symptom phenotype of symptomatic and asymptomatic plants of each cultivar 10 days after cut back.

Non‐transgenic FEC was produced from the CMD2‐type cultivars TME 7 and TME 3 to determine whether the observed loss of resistance was particular to TME 204. Micropropagated wild‐type and FEC‐derived plants obtained from TME 7 and TME 3 were challenged with EACMV‐K201, together with the corresponding lines of TME 204. The development of CMD was similar across all three cultivars, with the number of plants showing CMD symptoms and average symptom severity scores observed on FEC‐derived plants comparable with the values observed for the susceptible cultivar 60444. On inoculation with EACMV‐K201, 63%–90% of the wild‐type plants and FEC‐derived plants of all three cultivars developed disease symptoms. In all cases, wild‐type control plants then underwent recovery, with only a few plants (0%–22%) showing symptoms, whereas CMD within the FEC‐derived plants was maintained at a high level (63%–90%) throughout the study period (Fig. 4A). Similarly, CMD symptom severity scores of 2.6–3.5 for FEC‐derived and 1.7–2.6 for wild‐type plants were attained within 3–4 weeks after virus challenge. These then remained at high levels (3.0–5.0) in FEC‐derived plants of the three cultivars, whereas the severity scores of the wild‐type plants decreased to reach 0.0–0.6 by 15.5 weeks after inoculation (Fig. 4B,C).

**Figure 5:**
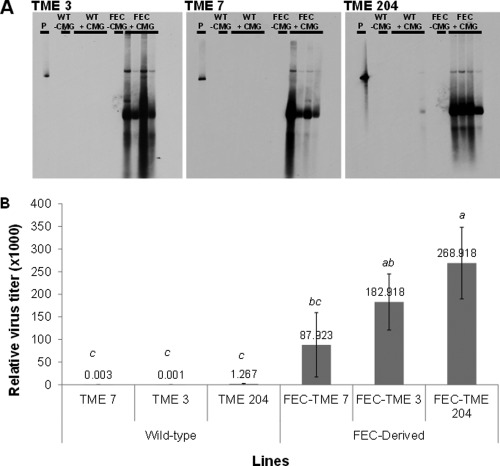
Virus titre of an infectious clone of *East African cassava mosaic virus* (EACMV‐K201) in wild‐type and friable embryogenic callus (FEC)‐derived plants of CMD2‐type cultivars. Virus titre determination by Southern blot (A) and quantitative polymerase chain reaction (qPCR) (B) performed on total DNA extracted from leaves of wild‐type and non‐recovering FEC‐derived plants at 108 days after inoculation (60 days after cut back). The primers used to label the probes for Southern blotting and for qPCR assay are shown in Table S1. Bars show standard error (*n* = 4); means followed by the same letter are not significantly (*P* ≤ 0.05) different. P, positive control; −CMG and +CMG denote without and with cassava mosaic geminivirus EACMV‐K201 inoculation, respectively.

The virus titres within fully expanded younger leaves of wild‐type and FEC‐derived plants of TME 204, TME 3 and TME 7 were assessed using both quantitative polymerase chain reaction (qPCR) and Southern blotting at 15.5 weeks after inoculation with EACMV‐K201 (8.5 weeks after cut back). Viral load was found to be significantly higher in the FEC‐derived plants of the three cultivars compared with the corresponding wild‐types, as shown by qPCR (Fig. 5B) and Southern blot (Fig. 5A) analysis for the presence of the DNA‐A component. With the exception of a low signal in one TME 204 plant, all four biological replicates from the wild‐types showed the presence of very low virus titres by qPCR, at a level undetectable by Southern blot.

### FEC‐derived plants are also susceptible to *African cassava mosaic virus* (ACMV)

The FEC‐derived plants of TME 204, TME 7 and TME 3 cultivars were challenged with *African cassava mosaic Cameroon virus* (ACMV‐CM) to assess their resistance to a less severe virus causing CMD. As expected, the number of plants showing CMD symptoms and the severity scores observed on plants challenged with ACMV were lower than those seen with EACMV‐K201. All wild‐type plants of TME 204 resisted the onset of CMD, whereas wild‐type TME 3 and TME 7 showed a smaller number of plants exhibiting CMD symptoms at 30% and 10%, respectively. All plants of these three cultivars then recovered to become symptom free by 35 days after challenge with ACMV, and remained so after cut back of the stems (Fig. S4A,B, see Supporting Information). In contrast, 90%–100% of FEC‐derived plants in all three cultivars developed CMD, and remained diseased with severity scores of 1.5–3.0, with minimal recovery observed (see Fig. S4A,B).

### Loss of resistance to CMD occurs in the early stages of somatic embryogenesis

**Figure 6:**
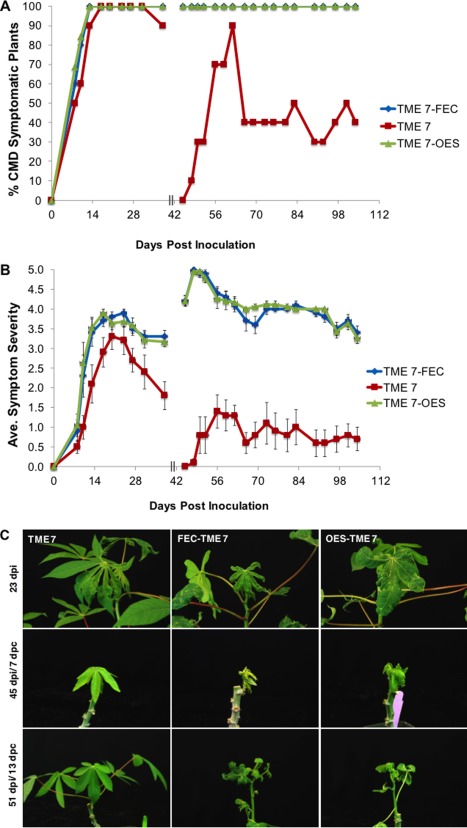
Response of TME 7 plants regenerated from friable embryogenic callus (FEC) and organized embryogenic structure (OES) tissues to inoculation with an infectious clone of *East African cassava mosaic virus* (EACMV‐K201) under glasshouse conditions. (A) Percentage of cassava mosaic disease (CMD) symptomatic plants. (B) Average symptom severity scores (scale 0–5). Plant stems were cut back at 38 days after biolistic inoculation and CMD was assessed on new leaf growth. Breaks in the *x* axis indicate a lapse in shoot regrowth after this cut back. Bars show standard error (*n* = 10–19). (C) CMD symptom phenotype of symptomatic and asymptomatic wild‐type, FEC‐derived and OES‐derived plants taken at 23, 45 and 51 days post‐inoculation (dpi) or at 7 and 13 days post cut‐back (dpc) where indicated.

In cassava, somatic embryogenesis is induced by the culture of leaf or axillary bud explants on Murashige and Skoog (MS) (Murashige and Skoog, 1962) or DKW/Juglans (Driver and Kuniyuki, 1984) basal medium supplemented with picloram (Chauhan *et al*., 2015; Taylor *et al*., 2012). The organized embryogenic structures (OESs) produced are then utilized to generate FEC by subculture onto Gresshoff and Doy (1974)‐based medium (Chauhan *et al*., 2015; Taylor *et al*., 1996). Alternatively, plants can be regenerated directly from OES by subculture onto medium containing cytokinins (Taylor *et al*., 2004). Twelve independent lines of TME 7 plants were recovered from OES (named ‘OES‐derived’ plants hereafter) and challenged with EACMV‐K201, together with FEC‐derived and micropropagated, wild‐type TME 7 plants. Observations of CMD symptom development and recovery over time showed that all (100%) TME 7 plant types developed CMD, with average symptom severity scores of 3.3–3.9 by 2–3 weeks after virus inoculation (Fig. 6A,B). As expected, wild‐type plants recovered from CMD and only 40% of the plants showed symptoms with an average score of 0.7 by 15 weeks after inoculation. In contrast, OES‐derived and FEC‐derived plants behaved in a similar manner to each other, maintaining a large number of plants (100%) with CMD symptoms and having severe to very severe symptoms (3.5–5.0) throughout the study period (Fig. 6A–C).

### Loss of resistance to CMD is stable in subsequent vegetative generations

The stability of the loss of resistance to CMD was assessed across generations of plants propagated via stake/stem cuttings. Third‐generation stake cuttings of TME 204 p5001‐expressing lines were generated in the glasshouse and inoculated with EACMV‐K201. All transgenic lines (100%) showed CMD symptoms with an average disease severity of 2.5–2.8 attained 3–4 weeks after challenge, compared with stake cuttings of the wild‐type controls which displayed 50% of plants with CMD symptoms and an average severity score of 1.0–1.2 at 5–6 weeks after challenge (Fig. S5A,B, see Supporting Information). Wild‐type plants recovered from CMD with only 16% showing symptoms and a very mild average severity score of 0.3. The p5001‐expressing lines did not undergo recovery from CMD and remained 100% symptomatic and had average severity scores of 2.5 by the end of the experiment at 16.5 weeks after virus challenge (Fig. S5A,B).

### FEC‐derived plants of a CMD1‐type cultivar retain the CMD recovery phenotype

**Figure 7:**
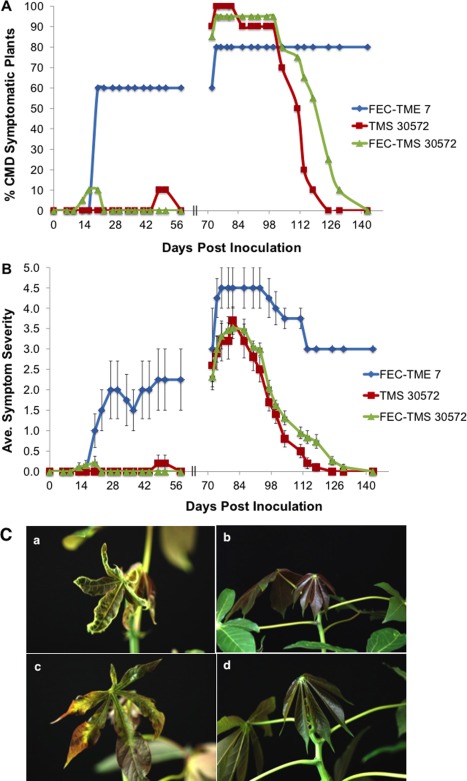
Response of friable embryogenic callus (FEC)‐derived and micropropagated wild‐type plants of CMD1‐type cultivar TMS 30572 to inoculation with an infectious clone of *East African cassava mosaic virus* (EACMV‐K201) under glasshouse conditions. (A) Percentage of cassava mosaic disease (CMD) symptomatic plants. (B) Average symptom severity scores (scale 0–5). Plants were first challenged with biolistic inoculation and, 30 days later, by grafting. Plant stems were cut back at 59 days after biolistic inoculation (29 days after grafting) and CMD was assessed on new leaf growth. Breaks in the *x* axis indicate a lapse in shoot regrowth after this cut back. Bars show standard error (*n* = 4–19). (C) Recovery from CMD in plants of TMS 30572: (a, c) CMD symptoms on wild‐type and FEC‐derived plants at 21 days after cut back; (b, d) recovery from CMD in wild‐type and FEC‐derived plants at 39 days after cut back.

FEC was produced and plants were regenerated from the CMD1‐type cultivar TMS 30572 to assess whether the induced susceptibility to CMD observed in CMD2‐type cultivars also occurred in cultivars possessing CMD1‐type resistance. Plants were established in the glasshouse and inoculated with EACMV‐K201 by microparticle bombardment. However, no CMD symptoms were seen to develop on FEC‐derived plant lines or micropropagated wild‐types of TMS 30572 by 4 weeks after inoculation. As an alternative challenge method, these plants were bud graft inoculated using scions from stock plants of a CMD‐susceptible cultivar infected with EACMV‐K201. Four weeks after grafting, all plants were cut back and CMD symptoms developed on new growth. By 6–7 weeks after bud grafting (1–2 weeks after cut back), 90%–100% of both wild‐type and FEC‐derived plants had developed CMD and showed average severity scores of 3.0–3.7 (Fig. 7A,B). Unlike the CMD2‐type cultivars described above, the FEC‐derived lines of TMS 30572 displayed recovery from CMD in a manner similar to the wild‐type plants, such that, by 8–10 weeks after cut back, new growth was seen to be free of CMD symptoms (Fig. 7A–C).

### FEC‐derived plants of the CMD3‐type cultivar TMS 98/0505 are resistant to CMD

**Figure 8:**
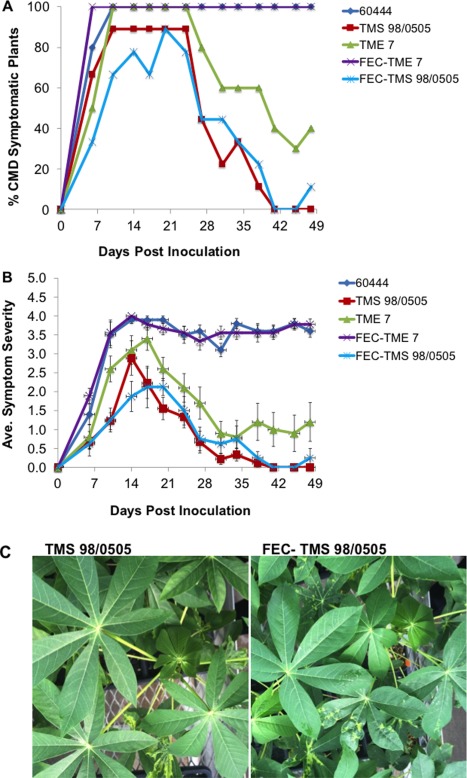
Response of friable embryogenic callus (FEC)‐derived and micropropagated wild‐type CMD3‐type cultivar TMS 98/0505 and CMD2‐type cultivar TME 7 plants to inoculation with an infectious clone of *East African cassava mosaic virus* (EACMV‐K201). (A) Percentage of cassava mosaic disease (CMD) symptomatic plants. (B) Average symptom severity scores (scale 0–5). Bars show standard error (*n* = 9–10). (C) Recovery from CMD seen in both wild‐type (left) and FEC‐derived (right) plants of TMS 98/0505 at 29 days post‐inoculation.

In a similar study, non‐transgenic plants were regenerated from the FEC‐derived CMD3‐type cultivar TMS 98/0505 and challenged with the infectious clone EACMV‐K201, together with wild‐type micropropagated TMS 98/0505, FEC‐derived and wild‐type TME 7 and the susceptible cultivar 60444. Maximum CMD symptom development and average symptom severity scores were recorded within 1–3 weeks after virus challenge, with each cultivar responding differently (Fig. 8A,B). The FEC‐derived 98/0505 and the micropropagated wild‐type control each showed a maximum of 88.9% CMD symptom development after challenge, with an average symptom severity score of 2.1–2.9 within 2–3 weeks after virus inoculation. After that time, new leaf growth was free of symptoms and plants recovered fully from disease by 6 weeks after inoculation, with no apparent difference between the FEC‐derived plant and wild‐type control (Fig. 8A–C). FEC‐derived and wild‐type plants of the CMD2‐type cultivar TME 7 and the susceptible control 60444 had CMD incidences of 100% and maximum average symptom severity scores of 3.4–4.0 within 1–3 weeks after virus inoculation (Fig. 8A,B). The wild‐type TME 7 showed gradual recovery, with only 40% of plants having CMD symptoms and an average severity score of 1.0, whereas plants of FEC‐derived TME 7 and 60444 maintained 100% CMD symptoms and average severity scores of 3.1–4.0 throughout the 6‐week study period (Fig. 8A,B).

## Discussion

We have reported previously proof of concept for RNAi‐mediated resistance to CBSD under field trial conditions at Namulonge, Uganda (Odipio *et al*., 2014; Ogwok *et al*., 2012). As the transgenic cassava lines described at that time were derived from the CMD‐susceptible cultivar 60444, the high incidence of CMD observed on these plants was expected. As the next step in the development of CBSD resistance germplasm (Taylor *et al*., 2012), the Ugandan farmer‐preferred cultivar TME 204 was modified with an improved RNAi construct (Chauhan *et al*., 2015) and field trialled at the same location. Cassava cultivars, such as TME 204, which possess the dominant monogenic CMD2‐type resistance, develop typical CMD symptoms on their leaves, but then recover from the disease with minimal suppression of storage root yields (Okogbenin *et al*., 2013). The data presented here show that the CMD2 resistance mechanism was negated in plants of transgenic lines of TME 204 cultivated under field conditions.

Uniform susceptibility to CMD across the p5001 transgenic field‐grown plants raised concerns that the loss of resistance to CMD resulted from the accumulation of transgene‐derived siRNAs generated by the CP sequences of CBSV and UCBSV. Such off‐target effects have been reported previously in some plant (Xu *et al*., 2006) and animal (Tschuch *et al*., 2008) systems. Data generated from glasshouse studies clearly demonstrated that loss of resistance to both ACMV and EACMV was not caused by the field environment (Figs 2A,B and S4A,B). These studies also showed that neither transgene‐derived siRNAs nor transgenes generally were the underlying cause for the loss of resistance to CMD (Figs 2A,B and S3).

Evidence showed that the passage of tissues, regardless of whether or not they contained transgene sequences, through somatic embryogenesis was sufficient to cause the resulting plants to become susceptible to CMD. These plants develop symptoms in a manner similar to the susceptible cultivar 60444 and are unable to undergo the recovery phenotype, with its associated reduction in disease symptoms and viral load, that characterizes CMD2‐type cultivars (Figs 4 and 5). Importantly, this phenomenon could be replicated in the two additional varieties TME 3 and TME 7, both of which have been characterized to possess a functional CMD2 locus (Akano *et al*., 2002; Lokko *et al*., 2005). No influence of the type of explant (leaf or axillary bud) or medium additives used to induce the embryogenic tissues (Chauhan *et al*., 2015) could be found. Instead, a simple 3–4‐week induction of somatic embryos (OES) on auxin‐containing medium was sufficient to cause all regenerated plants to lose CMD2‐type resistance. This acquired susceptibility was demonstrated to be stable over multiple cycles of clonal propagation in the glasshouse (Fig. S5A,B) and two generations under field conditions (data not shown), with no reversion to the CMD2‐type resistant phenotype observed to date.

All commonly used genetic transformation systems in cassava rely on the induction of somatic embryogenesis. In some cases, OES produced from explants is used directly for transgene integration (Ihemere *et al*., 2012; Ntui *et al*., 2015). Alternatively, OES can be matured to generate caulogenic cotyledon tissues (Jorgensen *et al*., 2005; Li *et al*., 1996), or converted to a highly disorganized FEC, which can then be subjected to *Agrobacterium* or direct gene transfer technologies (Liu *et al*., 2011; Taylor *et al*., 2004). FEC‐based methods are the most commonly employed and were utilized to produce the transgenic plants studied here (Chauhan *et al*., 2015). Somaclonal variation within cassava plants regenerated from FEC has been reported previously, but was associated with a subset of the regenerants that showed distinct off‐type morphology, characterized by weak stem growth and epinastic leaf curling (Raemakers *et al*., 1995; Taylor *et al*., 2001). The culture‐induced loss of resistance to CMD described here differs from these earlier observations. In the present case, 100% of the regenerated CMD2‐type plants exhibited uniform loss of resistance to geminivirus pathogens, but were otherwise phenotypically indistinguishable from the CMD‐resistant mother plants from which they were derived. This highly reproducible and stable change took place during the production of OES (Fig. 6A–C) prior to its conversion to FEC. The culture of rapidly dividing, highly disorganized callus tissues, such as the FEC used in cassava transformation systems, is known to induce changes at the genetic and epigenetic levels (Kaeppler *et al*., 2000; Ma *et al*., 2015; Miguel and Marum, 2011). However, it was not expected that such changes should occur within the OES, a tissue type that maintains highly coordinated cell divisions throughout the morphogenic and regeneration processes. A recent study on cassava has also confirmed that decreases in DNA methylation occur in continuously cultured FEC, but not in somatic embryos (Ma *et al*., 2015). To our knowledge, loss of an essential trait in this manner is unprecedented in the literature.

Vanderschuren *et al*. (2012) reported that, under glasshouse conditions, in FEC‐derived TME 7 transgenic CBSD‐resistant cassava plants inoculated with *East African cassava mosaic virus* – Uganda (EACMV‐Ug) alone or in combination with CBSV using a top‐grafting method, TME 7 scions developed typical mosaic symptoms on the first emerging leaves, followed by a recovery from the symptoms. However, these researchers have subsequently also observed a loss of CMD resistance in transgenic TME 7 plants engineered for CBSD resistance under field conditions (H. Vanderschuren and W. Gruissem, personal communication). At this time, the causal genetic and molecular mechanisms underlying the induced loss of resistance to CMD remain unknown. Somaclonal variation has been shown to result from both genetic and epigenetic changes (Ji *et al*., 2015; Miyao *et al*., 2012; Ong‐Abdullah *et al*., 2015; Stroud *et al*., 2013). The ‘mantled’ phenotype observed in African oil palm is a well‐documented example of a somaclonal variant that was recently confirmed to be a result of DNA hypomethylation (Ong‐Abdullah *et al*., 2015). Decreases in DNA methylation have been reported to occur in continuously cultured FEC, but not in somatic embryos (OES) (Ma *et al*., 2015). However, the uniformity of the culture‐induced change described here for CMD is consistent with epigenetic changes that occur during the induction of OES. This could be at the CMD2 locus itself (*cis*‐) or elsewhere in the genome (*in trans*‐) that affects the functionality of CMD2. This change appears to be highly stable, as susceptibility to CMD is maintained after multiple cycles of vegetative propagation. The stability of epigenetic changes in clonal propagation (Jaligot *et al*., 2011) and through the reproductive cycle (Stelpflug *et al*., 2014; Stroud *et al*., 2013) has been reported in other species. Efforts are therefore ongoing to determine whether culture‐induced loss of resistance to CMD in cassava is maintained through meiosis and into the subsequent progeny.

TME 3 and TME 7 were collected from farmers’ fields in Nigeria and are very closely related. TME 204 reportedly originates from Benin (Lokko *et al*., 2006), is genetically distinct from the TME 3 types and is closely related to TME 419 (Rabbi *et al*., 2014). The results reported here indicate high probability that the two groups possess similar CMD2‐type resistance mechanisms, and that all such cultivars will be compromised for resistance to CMD when passed through somatic embryogenesis. CMD2‐type cultivars have been widely adopted throughout East, West and Central Africa and perform well against CMD. However, all are highly susceptible to CBSD and are constrained for other traits common to cassava.

Importantly, the loss of CMD resistance through somatic embryogenesis appears to be specific to CMD2‐type cultivars, and does not occur in CMD1‐ or CMD3‐type cultivars. Plants of the CMD1‐type cultivar TMS 30572 and CMD3‐type cultivar TMS 98/0505, regenerated through somatic embryogenesis, retained their CMD recovery phenotype (Figs 7 and 8). Although this result requires verification under field conditions, where plants are exposed to natural infection by different species of CMG, it indicates that CMD1‐ and CMD3‐type cultivars could be transgenically enhanced using existing culture systems. Similar efforts directed at CMD2 types, however, will most probably require the development of new regeneration systems that are not reliant on somatic embryogenesis.

CMD2‐type cultivars are widely grown by farmers in Africa and commonly employed in breeding programmes (Kawuki *et al*., 2011; Okogbenin *et al*., 2013). Although no verified reports exist of CMD2 resistance breakdown in the field, reliance on monogenic resistance strategies is known to carry risk. The observations described in the present report clearly show that CMD2‐mediated resistance can break down under certain circumstances, and indicate the potential vulnerability of this resistance mechanism. More than 100 million people across tropical Africa presently rely on the functionality of CMD2 to secure their daily calories against the threat of CMD. It is essential that efforts are focused on gaining a full understanding of its underlying molecular mechanisms. The first of such studies has recently been published describing the methylome of TME 7, a CMD2‐type cultivar (Wang *et al*., 2015), which will be instrumental in understanding the somatic embryogenesis‐induced changes described above.

The results presented in this study strengthen the rationale of recent and ongoing strategies for stacking CMD1, CMD2 and CMD3 resistance mechanisms within the same genetic background (Okogbenin *et al*., 2013) to ensure durable resistance to CMD.

## Experimental Procedures

### Plasmid constructs used for *Agrobacterium*‐mediated transformation

Transgenic cassava plants were generated using constructs p718 (Yadav *et al*., 2011), p5001 (Chauhan *et al*., 2015), p5003 and pRS26. Expression cassettes of these constructs were generated in pCAMBIA2300 (Genbank: AF234315) modified by the removal of the LacZ open reading frame and insertion of Multi‐Left‐Border sequences (PureMlb™ technology from Japan Tobacco, Iwata, Shizuoka, Japan) (Kuraya *et al*., 2004). Constructs p718, p5001 and p5003 consisted of inverted repeat sequences from the CP genes of UCBSV (p718), CBSV (p5003) and both CPs (p5001). pRS26 was designed to elevate iron content in cassava storage roots by expression of the *FEA1* gene under control of the Patatin type‐1 promoter (Ihemere *et al*., 2012).

### Production of embryogenic tissues, genetic transformation and plant regeneration

Plants of African cassava cultivars TMS 60444, TME 7, TME 3, TME 204, TMS 30572 and TMS 98/0505 were micropropagated and maintained *in vitro* as described previously (Taylor *et al*., 2012). OESs were induced from immature leaf and axillary bud explants by culture on MS basal medium (Murashige and Skoog, 1962) or DKW/Juglans basal salts (Driver and Kuniyuki, 1984) plus MS vitamins supplemented with 50 μm picloram. OES was subcultured onto Gresshoff and Doy basal medium (Gresshoff and Doy, 1972) containing 50 μm picloram and various supplements for the production of FEC (Chauhan *et al*., 2015). FEC was used as target tissue for *Agrobacterium*‐mediated genetic transformation and recovery of transgenic plants, as described previously (Chauhan *et al*., 2015). Five different types of *in vitro*‐derived plant were produced and studied: (i) plants propagated only by shoot multiplication on MS medium and not subjected to somatic embryogenic processes (wild‐type); (ii) plants confirmed to be transgenic for one of the four constructs described above; (iii) plants which had been through the transformation system, but confirmed not to have integrated T‐DNA (escapes); (iv) plants regenerated from FEC without passing through the transformation system (FEC‐derived); and (v) plants regenerated from OES tissues by culture of this tissue on MS basal medium supplemented with cytokinin (OES‐derived).

### Molecular characterization of transgenic plants

The characterization of transgenic *in vitro* plantlets of p5001 for transgene presence and copy number using dot blots has been described elsewhere (Chauhan *et al*., 2015). *In vitro* plantlets of putative transgenic lines of p5003, p718 and pRS26 were first tested for expression of the selectable marker gene using the NPTII ImmunoStrip test according to the manufacturer's protocol (Agdia, Elkhart, IN, USA). Positive lines were further tested for the presence and expression of the trait gene(s) using PCR and reverse transcription (RT)‐PCR (for over‐expression), respectively, with the primers listed in Table S1 (see Supporting Information). The detection of small RNA from transgenic lines generated using inverted repeat constructs p5001, p718 and p5003 was performed as described elsewhere (Yadav *et al*., 2011). The transgenic lines were then transferred to soil and established in the glasshouse.

### Plant establishment and growth in the glasshouse

*In vitro* plantlets were transferred to Fafard 51 soil‐less potting medium (Conrad Fafard, Inc., Agawam, MA, USA) in 3‐in pots and weaned under 100% humidity on a mist bench for 7 days (Taylor *et al*., 2012). Plants were then transferred to the open bench and grown at a temperature of 26 ºC/25 ºC (day/night) with 60%–90% relative humidity for 3 weeks to reach approximately 8 cm in height, at which time they were transferred to a glasshouse maintained at a temperature of 32 ºC/27 ºC (day/night) and 70%–95% relative humidity. For propagation by stake cutting, stakes of 12–20 cm in length possessing four to six nodes were prepared from the mature semi‐woody portion of 4–6‐month‐old glasshouse‐grown plants and established in 4‐in pots filled with potting medium as described above.

### Geminivirus inoculation and assessment of CMD symptoms in the glasshouse

Four‐week‐old glasshouse‐grown plants, approximately 10 cm in height, were inoculated with infectious clones of EACMV‐K201 (EACMV‐KE[KE:Msa:K201:02], DNA‐A GenBank: AJ717541 and DNA‐B GenBank: AJ704953) (Bull *et al*., 2006; Patil and Fauquet, 2010) and ACMV‐CM (ACMV‐[CM:DO2:98], DNA‐A GenBank: AF112352 and DNA‐B GenBank: AF112353) (Fondong *et al*., 2000) using a Helios® Gene Gun (BioRad, Hercules, CA, USA). Plasmid DNA harbouring the viral infectious clones was extracted from overnight culture using a Qiagen Maxiprep kit (Qiagen, Hilden, Germany). Gold microparticles of 1 μm in size (Inbio Gold, Hurstbridge, Vic., Australia) were coated with 50 ng/µL DNA‐A and DNA‐B plasmids of infectious clone ACMV‐CM, and 10 ng/µL DNA‐A and DNA‐B plasmids of EACMV‐K201. To prepare 50 virus inoculation shots, 25 mg of gold particles were washed with 100% ethanol, sonicated and vortexed until completely disaggregated, and then washed and resuspended in sterile nuclease‐free H_2_O. Plasmid DNA was added dropwise to the gold suspension whilst vortexing, followed by 100 µL of 0.5 m spermidine and 100 µL of 1.0 m CaCl_2_. The supernatant was removed and DNA‐coated gold particles were resuspended and washed three times in 100% ethanol before final resuspension in 3.5 mL of 100% ethanol containing 0.1 mg/mL polyvinylpyrrolidone (PVP 36000; Sigma‐Aldrich, St. Louis, MO, USA). A 75‐cm length of Tefzel tubing (Inbio Gold) was coated with the DNA/gold suspension according to the BioRad Helios protocol (Bio‐Rad Bulletin 9541, http://www.docstip.com/file/helios-gene-gun-system-instruction-manual.html). Coated tubing was cut into 1.27‐cm lengths to generate individual ‘shots’ carrying approximately 100 ng of viral DNA for ACMV‐CM and 20 ng for EACMV‐K201. A 5 × 5‐cm^2^ cardboard shield was used to cover and protect the meristem and youngest, still folded leaf, and each plant was shot once at 270 psi by gently pressing the Helios® Gene Gun nozzle against the first unfolded leaf where the veins converged.

Each inoculation experiment was performed at least twice. Individual plants were assessed for the development of CMD symptoms starting 7–15 days after inoculation and every 3–7 days thereafter. Plants were scored visually based on a scale of 0–5 (Fauquet and Fargette, 1990), where ‘0’ represents no disease, ‘1’ mild mosaic with no leaf distortion, ‘2’ moderate mosaic with slight leaf distortion, ‘3’ moderate mosaic with distinct leaf malformation, ‘4’ severe mosaic with severe leaf formation and ‘5’ very severe mosaic with complete leaf deformation. Plant stems were cut back to approximately 12.5 cm above the soil level to remove all leaves at 5–9 weeks after inoculation. The development of CMD symptoms was assessed on new growth starting 5–10 days after cut back and every 3–7 days thereafter using the same 0–5 visual scale as described above.

Graft inoculation was performed using the chip bud method as described previously (Wagaba *et al*., 2013). Eight‐ to ten‐week‐old plants of the CMD‐susceptible cultivar TME 14 infected with EACMV‐K201 were used as the source of scions for the transmission of geminivirus to test materials.

### Virus titre determination

Genomic DNA was extracted from the first fully expanded leaf of glasshouse‐grown cassava plants following the cetyltrimethylammonium bromide (CTAB) protocol (Doyle and Doyle, 1990), as described previously (Chauhan *et al*., 2015). Samples were treated with DNase‐free RNAseA according to the manufacturer's instructions (Roche, Indianapolis, IN, USA) and the genomic DNA was used for virus titre determination by qPCR and Southern blot analysis. For EACMV‐K201 virus titre determination, qPCR was run following the method described by Ogwok *et al*. (2015) using genomic DNA as a template. The primers used for qPCR and for the labelling of probes employed for Southern blot are listed in Table S1. Genomic Southern blotting was performed using 2 μg of undigested genomic DNA, as described by Chauhan *et al*. (2015). The probes used for detection were amplified from DNA‐A of EACMV‐K201 using the primers indicated in Table S1.

### Establishment and assessment of transgenic TME 204 plants under field conditions

Twenty‐five independent p5001 transgenic TME 204 plant lines and wild‐type controls were established in 50‐mL Falcon tubes (Ogwok *et al*., 2012), transported to the National Crops Resources Research Institute (NaCRRI), Namulonge, Uganda, and established under CFT conditions. The trial was established in a randomized complete block design with eight replicates of each plot containing 10 plants. Each plant in the trial was scored for the incidence of CMD. Scoring for symptom expression was carried out monthly beginning at 4 weeks after establishment in the field until final harvest at 12 MAP.

## Supporting information

Additional Supporting Information may be found in the online version of this article at the publisher's website:

**Fig. S1** Incidence of cassava mosaic disease (CMD) in RNA interference (RNAi) p5001 TME 204 lines under confined field trial (CFT) conditions at NaCRRI, Namulonge, Uganda. p5001 lines express an inverted repeat of coat protein genes derived from *Ugandan cassava brown streak virus* and *Cassava brown streak virus* fused in tandem. CMD incidence shown at 12 months after planting. Data were collected from five replicates (10 plants each) per line.

**Fig. S2** Detection of transgenes in TME 204 transgenic plants using polymerase chain reaction (PCR). Transgenic lines expressing the inverted repeat of the coat protein gene derived from *Ugandan cassava brown streak virus* (p718, A) and *Cassava brown streak virus* (p5003, B) and transgenic lines expressing the iron assimilatory protein gene *FEA1* (pRS26, C) were generated and confirmed for the presence of the transgene using the primer pairs described in Table S1 before challenge with an infectious clone of *East African cassava mosaic virus* (EACMV‐K201) under glasshouse conditions. Positive controls are PCR products using plasmid DNA of the respective constructs as a template.

**Fig. S3** Response of transgenic TME 204 lines to biolistic inoculation with an infectious clone of *East African cassava mosaic virus* (EACMV‐K201) under glasshouse conditions. Transgenic lines expressing the inverted repeat of coat protein genes derived from *Ugandan cassava brown streak virus* (UCBSV‐CP) and *Cassava brown streak virus* (CBSV‐CP) fused in tandem (p5001), transgenic lines expressing the inverted repeat of CBSV‐CP (p5003) or UCBSV‐CP (p718), transgenic lines expressing the iron assimilatory protein gene *FEA1* (pRS26) and susceptible control cv. 60444 were used. We observed the typical recovery phenotype in infected wild‐type TME 204 and non‐recovery phenotype in transgenic (susceptible lines) lines 9 weeks after virus challenge.

**Fig. S4** Response of friable embryogenic callus (FEC)‐derived and micropropagated wild‐type plants of CMD2‐type cultivars to inoculation with *African cassava mosaic Cameroon virus* (ACMV‐CM) under glasshouse conditions. FECs were generated and plants were recovered from TME 3, TME 7 and TME 204. (A) Percentage of cassava mosaic disease (CMD) symptomatic plants. (B) Average symptom severity scores (scale of 0–5). Plant stems were cut back at 41 days after biolistic inoculation and CMD was assessed on new leaf growth. Breaks in the *x* axis indicate a lapse in shoot regrowth after this cut back. Bars show standard error (*n* = 9–10).

**Fig. S5** Response of transgenic p5001 TME 204 lines from third‐generation stake cuttings to biolistic inoculation with a modified infectious clone of *East African cassava mosaic virus* (EACMV‐K201) under glasshouse conditions. Percentage of cassava mosaic disease (CMD) symptomatic plants (A) and average symptom severity scores (scale of 0–5) (B) of transgenic lines expressing the inverted repeat of coat protein genes derived from *Ugandan cassava brown streak virus* and *Cassava brown streak virus* fused in tandem (p5001, four lines) and micropropagated wild‐type TME 204. Plant stems were cut back at 55 days after biolistic inoculation and CMD was assessed on new leaf growth. Breaks in the *x* axis indicate a lapse in shoot regrowth after this cut back. Bars show standard error (*n* = 6). The infectious clone was modified to contain 452 bp of green fluorescent protein (GFP) coding sequence that replaced the same length of the coat protein gene of EACMV‐K201.

**Table S1** List of primers used for transgene detection, labelling of probes and virus titre determination using quantitative polymerase chain reaction (qPCR).

## References

[mpp12353-bib-0001] Abaca, A. , Kawuki, R. , Tukamuhabwa, P. , Baguma, Y. , Pariyo, A. , Alicai, T. , Omongo, C. and Bua, A. (2012) Evaluation of local and elite cassava genotypes for resistance to cassava brown streak disease in Uganda. J. Agron. 11, 65–72.

[mpp12353-bib-0002] Akano, O. , Dixon, O. , Mba, C. , Barrera, E. and Fregene, M. (2002) Genetic mapping of a dominant gene conferring resistance to cassava mosaic disease. Theor. Appl. Genet. 105, 521–525. 12582500 10.1007/s00122-002-0891-7

[mpp12353-bib-0003] Bull, S.E. , Briddon, R.W. , Sserubombwe, W.S. , Ngugi, K. , Markham, P.G. and Stanley, J. (2006) Genetic diversity and phylogeography of cassava mosaic viruses in Kenya. J. Gen. Virol. 87, 3053–3065. 16963765 10.1099/vir.0.82013-0

[mpp12353-bib-0004] Bull, S. , Owiti, J. , Niklaus, M. , Beeching, J.R. , Gruissem, W. and Vanderschuren, H. (2009) Agrobacterium‐mediated transformation of friable embryogenic calli and regeneration of transgenic cassava. Nat. Protoc. 4, 1845–1854. 20010938 10.1038/nprot.2009.208

[mpp12353-bib-0005] Chauhan, R.D. , Beyene, G. , Kalyaeva, M. , Fauquet, C.M. and Taylor, N. (2015) Improvements in Agrobacterium‐mediated transformation of cassava (*Manihot esculenta* Crantz) for large‐scale production of transgenic plants. Plant Cell Tiss. Org. 121, 591–603.

[mpp12353-bib-0006] Doyle, J.J. and Doyle, J.L. (1990) Isolation of plant DNA from fresh tissue. Focus, 12, 13–15.

[mpp12353-bib-0007] Driver, J.A. and Kuniyuki, A.H. (1984) *In vitro* propagation of paradox walnut rootstock. Hort Sci. 19, 507–509.

[mpp12353-bib-0008] Fauquet, C. and Fargette, D. (1990) African cassava mosaic virus: etiology, epidemiology and control. Plant Dis. 74, 404–411.

[mpp12353-bib-0009] Fondong, V.N. , Pita, J.S. , Rey, M.E. , de Kochko, A. , Beachy, R.N. and Fauquet, C.M. (2000) Evidence of synergism between African cassava mosaic virus and a new double‐recombinant geminivirus infecting cassava in Cameroon. J. Gen. Virol. 81, 287–297. 10640569 10.1099/0022-1317-81-1-287

[mpp12353-bib-0010] Fregene, M. and Puonti‐Kaerlas, J. (2002) Cassava biotechnology. In: Cassava: Biology, Production and Utilization (Hillocks R.J. and Thresh J.M., eds), pp. 179–208. Wallingford, CT: CABI

[mpp12353-bib-0011] Gresshoff, P.M. and Doy, C.H. (1972) Development and differentiation of haploid *Lycopersicon esculentum* (tomato). Planta, 107, 161–170. 24477400 10.1007/BF00387721

[mpp12353-bib-0012] Gresshoff, P.M. and Doy, C.H. (1974) Derivation of a haploid cell line from *Vitis vinifera* and the importance of the stage of meiotic development of the anthers for haploid culture of this and other genera. Zt. Pflanzenphysiol. 73, 132–141.

[mpp12353-bib-0013] Hahn, S.K. , Terry, E.R. and K. Leuschner, K. (1980) Breeding cassava for resistance to cassava mosaic disease. Euphytica, 29, 673–683.

[mpp12353-bib-0014] Hillocks, R.J. and Thresh, J.M. (2000) Cassava mosaic and cassava brown streak virus diseases in Africa: a comparative guide to symptoms and aetiologies. Roots, 7, 1–8.

[mpp12353-bib-0015] Ihemere, U.E. , Narayanan, N.N. and Sayre, R.T. (2012) Iron biofortification and homeostasis in transgenic cassava roots expressing the algal iron assimilatory gene, FEA1. Front. Plant Sci. 3, 171. 22993514 10.3389/fpls.2012.00171 PMC3440605

[mpp12353-bib-0016] IITA (1990) Annual Report (International Institute of Tropical Agriculture). Ibadan Nigeria.

[mpp12353-bib-0017] Jaligot, E. , Adler, S. , Debladis, E. , Beule, T. , Richaud, F. , Ilbert, P. , Finnegan, E.J. and Rival, A. (2011) Epigenetic imbalance and the floral developmental abnormality of the in vitro‐regenerated oil palm *Elaeis guineensis* . Ann. Bot. 108, 1453–1462. 21224269 10.1093/aob/mcq266 PMC3219487

[mpp12353-bib-0018] Ji, L. , Neumann, Drexel A. and Schmitz, Robert J. (2015) Crop epigenomics: identifying, unlocking, and harnessing cryptic variation in crop genomes. Mol. Plant, 8, 860–870. 25638564 10.1016/j.molp.2015.01.021 PMC5121661

[mpp12353-bib-0019] Jorgensen, K. , Bak, S. , Busk, P.K. , Sorensen, C. , Olsen, C.E. , Puonti‐Kaerlas, J. and Moller, B.L. (2005) Cassava plants with a depleted cyanogenic glucoside content in leaves and tubers. Distribution of cyanogenic glucosides, their site of synthesis and transport, and blockage of the biosynthesis by RNA interference technology. Plant Physiol. 139, 363–374. 16126856 10.1104/pp.105.065904 PMC1203385

[mpp12353-bib-0020] Kaeppler, S.M. , Kaeppler, H.F. and Rhee, Y. (2000) Epigenetic aspects of somaclonal variation in plants. Plant Mol. Biol. 43, 179–188. 10999403 10.1023/a:1006423110134

[mpp12353-bib-0021] Kaweesi, T. , Kawuki, R. , Kyaligonza, V. , Baguma, Y. , Tusiime, G. and Ferguson, M.E. (2014) Field evaluation of selected cassava genotypes for cassava brown streak disease based on symptom expression and virus load. Virol. J. 11, 216. 25526680 10.1186/s12985-014-0216-x PMC4304613

[mpp12353-bib-0022] Kawuki, R.S. , Pariyo, A. , Amuge, T. , Nuwamanya, E. , Ssemakula, G. , Tumwesigye, S. , Bua, A. , Baguma, Y. , Omongo, C. and Alicai, T. (2011) A breeding scheme for local adoption of cassava (*Manihot esculenta* Crantz). J. Plant Breed. Crop Sci. 3, 120–130.

[mpp12353-bib-0023] Kuraya, Y. , Ohta, S. , Fukuda, M. , Hiei, Y. , Murai, N. , Hamada, K. , Ueki, J. , Imaseki, H. and Komari, T. (2004) Suppression of transfer of non‐T‐DNA ‘vector backbone’ sequences by multiple left border repeats in vectors for transformation of higher plants mediated by *Agrobacterium tumefaciens* . Mol. Breed. 14, 309–320.

[mpp12353-bib-0024] Legg, J.P. (1999) Emergence, spread and strategies for controlling the pandemic of cassava mosaic virus disease in east and central Africa. Crop Protect. 18, 627–637.

[mpp12353-bib-0025] Legg, J.P. , Owor, B. , Sseruwagi, P. and Ndunguru, J. (2006) Cassava mosaic virus disease in East and Central Africa: epidemiology and management of a regional pandemic. Adv. Virus Res. 67, 355–418. 17027685 10.1016/S0065-3527(06)67010-3

[mpp12353-bib-0026] Legg, J.P. , Jeremiah, S.C. , Obiero, H.M. , Maruthi, M.N. , Ndyetabula, I. , Okao‐Okuja, G. , Bouwmeester, H. , Bigirimana, S. , Tata‐Hangy, W. and Gashaka, G. (2011) Comparing the regional epidemiology of the cassava mosaic and cassava brown streak virus pandemics in Africa. Virus Res. 159, 161–170. 21549776 10.1016/j.virusres.2011.04.018

[mpp12353-bib-0027] Li, H.Q. , Sautter, C. , Potrykus, I. and Puonti‐Kaerlas, J. (1996) Genetic transformation of cassava (*Manihot esculenta* Crantz). Nat. Biotechnol. 14, 736–740. 9630981 10.1038/nbt0696-736

[mpp12353-bib-0028] Liu, J. , Zheng, Q. , Ma, Q. , Gadidasu, K.K. and Zhang, P. (2011) Cassava genetic transformation and its application in breeding. J. Integr. Plant Biol. 53, 552–569. 21564542 10.1111/j.1744-7909.2011.01048.x

[mpp12353-bib-0029] Lokko, Y. , Danquah, E.Y. , Offei, S.K. , Dixon, A.G.O. and Gedil, M.A. (2005) Molecular markers associated with a new source of resistance to the cassava mosaic disease. Afr. J. Biotechnol. 4, 873–881.

[mpp12353-bib-0030] Lokko, Y. , Dixon, A. , Offei, S. , Danquah, E. and Fregene, M. (2006) Assessment of genetic diversity among African cassava *Manihot esculenta* Grantz accessions resistant to the cassava mosaic virus disease using SSR markers. Genet. Resour. Crop Ev. 53, 1441–1453.

[mpp12353-bib-0031] Ma, Q. , Zhou, W. and Zhang, P. (2015) Transition from somatic embryo to friable embryogenic callus in cassava: dynamic changes in cellular structure, physiological status, and gene expression profiles. Front. Plant Sci. 6, 824. 26500668 10.3389/fpls.2015.00824 PMC4594424

[mpp12353-bib-0032] Miguel, C. and Marum, L. (2011) An epigenetic view of plant cells cultured in vitro: somaclonal variation and beyond. J. Exp. Bot. 62, 3713–3725. 21617249 10.1093/jxb/err155

[mpp12353-bib-0033] Miyao, A. , Nakagome, M. , Ohnuma, T. , Yamagata, H. , Kanamori, H. , Katayose, Y. , Takahashi, A. , Matsumoto, T. and Hirochika, H. (2012) Molecular spectrum of somaclonal variation in regenerated rice revealed by whole‐genome sequencing. Plant Cell Physiol. 53, 256–264. 22156226 10.1093/pcp/pcr172

[mpp12353-bib-0034] Monger, W. , Seal, S. , Isaac, A. and Foster, G. (2001) Molecular characterization of the Cassava brown streak virus coat protein. Plant Pathol. 50, 527–534.

[mpp12353-bib-0035] Murashige, T. and Skoog, F. (1962) A revised medium for rapid growth and bio assays with tobacco tissue cultures. Physiol. Plant. 15, 473–497.

[mpp12353-bib-0036] Nichols, R. (1947) Breeding cassava for virus resistance. East Afr. Agric. J. 12, 184–194.

[mpp12353-bib-0037] Ntui, V.O. , Kong, K. , Khan, R.S. , Igawa, T. , Janavi, G.J. , Rabindran, R. , Nakamura, I. and Mii, M. (2015) Resistance to Sri Lankan cassava mosaic virus (SLCMV) in genetically engineered cassava cv. KU50 through RNA silencing. PLoS One, 10, e0120551. 25901740 10.1371/journal.pone.0120551 PMC4406713

[mpp12353-bib-0038] Odipio, J. , Ogwok, E. , Taylor, N.J. , Halsey, M. , Bua, A. , Fauquet, C.M. and Alicai, T. (2014) RNAi‐derived field resistance to Cassava brown streak disease persists across the vegetative cropping cycle. GM Crops Food, 5, 16–19. 24296511 10.4161/gmcr.26408 PMC5033198

[mpp12353-bib-0039] Ogwok, E. , Odipio, J. , Halsey, M. , Gaitan‐Solis, E. , Bua, A. , Taylor, N.J. , Fauquet, C.M. and Alicai, T. (2012) Transgenic RNA interference (RNAi)‐derived field resistance to cassava brown streak disease. Mol. Plant Pathol. 13, 1019–1031. 22845735 10.1111/j.1364-3703.2012.00812.x PMC6638741

[mpp12353-bib-0040] Ogwok, E. , Alicai, T. , Rey, M.E.C. , Beyene, G. and Taylor, N.J. (2015) Distribution and accumulation of cassava brown streak viruses within infected cassava (*Manihot esculenta*) plants. Plant Pathol. 64, 1235–1246.

[mpp12353-bib-0041] Okogbenin, E. , Egesi, C.N. , Olasanmi, B. , Ogundapo, O. , Kahya, S. , Hurtado, P. , Marin, J. , Akinbo, O. , Mba, C. , Gomez, H. , de Vicente, C. , Baiyeri, S. , Uguru, M. , Ewa, F. and Fregene, M. (2012) Molecular marker analysis and validation of resistance to cassava mosaic disease in elite cassava genotypes in Nigeria. Crop Sci. 52, 2576–2586.

[mpp12353-bib-0042] Okogbenin, E. , Moreno, I. , Tomkins, J. , Fauquet, C.M. , Mkamilo, G. and Fregene, M. (2013) Marker‐assisted breeding for cassava mosaic disease resistance. In: *Translational Genomics for Crop Breeding: Biotic Stress* (Varshney R and Tuberosa R., eds), Vol. 1, pp. 291–325. Chichester: Wiley.

[mpp12353-bib-0043] Ong‐Abdullah, M. , Ordway, J.M. , Jiang, N. , Ooi, S.E. , Kok, S.Y. , Sarpan, N. , Azimi, N. , Hashim, A.T. , Ishak, Z. , Rosli, S.K. , Malike, F.A. , Bakar, N.A. , Marjuni, M. , Abdullah, N. , Yaakub, Z. , Amiruddin, M.D. , Nookiah, R. , Singh, R. , Low, E.T. , Chan, K.L. , Azizi, N. , Smith, S.W. , Bacher, B. , Budiman, M.A. , Van Brunt, A. , Wischmeyer, C. , Beil, M. , Hogan, M. , Lakey, N. , Lim, C.C. , Arulandoo, X. , Wong, C.K. , Choo, C.N. , Wong, W.C. , Kwan, Y.Y. , Alwee, S.S. , Sambanthamurthi, R. and Martienssen, R.A. (2015) Loss of Karma transposon methylation underlies the mantled somaclonal variant of oil palm. Nature, 525, 533–537. 26352475 10.1038/nature15365 PMC4857894

[mpp12353-bib-0044] Patil, B.L. and Fauquet, C.M. (2010) Differential interaction between cassava mosaic geminiviruses and geminivirus satellites. J. Gen. Virol. 91, 1871–1882. 20335493 10.1099/vir.0.019513-0

[mpp12353-bib-0045] Patil, B.L. , Legg, J.P. , Kanju, E. , and Fauquet, C.M. (2015) Cassava brown streak disease. A threat to food security in Africa. J. Gen Virol., 96, 956–968. 26015320 10.1099/vir.0.000014

[mpp12353-bib-0046] Rabbi, I.Y. , Hamblin, M.T. , Kumar, P.L. , Gedil, M.A. , Ikpan, A.S. , Jannink, J.‐L. and Kulakow, P.A. (2014) High‐resolution mapping of resistance to cassava mosaic geminiviruses in cassava using genotyping‐by‐sequencing and its implications for breeding. Virus Res. 186, 87–96. 24389096 10.1016/j.virusres.2013.12.028

[mpp12353-bib-0047] Raemakers, C. , Jacobsen, E. and Visser, R. (1995) Histology of somatic embryogenesis and evaluation of somaclonal variation. In: *Cassava Biotechnology Network: Proceedings of the 2nd International Scientific Meeting*, pp. 336–354. CIAT Working Document 150. CIAT, Cali, Colombia.

[mpp12353-bib-0048] Scholthof, K.B. , Adkins, S. , Czosnek, H. , Palukaitis, P. , Jacquot, E. , Hohn, T. , Hohn, B. , Saunders, K. , Candresse, T. , Ahlquist, P. , Hemenway, C. and Foster, G.D. (2011) Top 10 plant viruses in molecular plant pathology. Mol. Plant Pathol. 12, 938–954. 22017770 10.1111/j.1364-3703.2011.00752.x PMC6640423

[mpp12353-bib-0049] Stelpflug, S.C. , Eichten, S.R. , Hermanson, P.J. , Springer, N.M. and Kaeppler, S.M. (2014) Consistent and heritable alterations of DNA methylation are induced by tissue culture in maize. Genetics, 198, 209–218. 25023398 10.1534/genetics.114.165480 PMC4174933

[mpp12353-bib-0050] Stroud, H. , Ding, B. , Simon, S.A. , Feng, S. , Bellizzi, M. , Pellegrini, M. , Wang, G.L. , Meyers, B.C. and Jacobsen, S.E. (2013) Plants regenerated from tissue culture contain stable epigenome changes in rice. Elife, 2, e00354. 23539454 10.7554/eLife.00354 PMC3601819

[mpp12353-bib-0051] Taylor, N.J. , Edwards, M. , Kiernan, R.J. , Davey, C.D.M. , Blakesley, D. and Henshaw, G.G. (1996) Development of friable embryogenic callus and suspension culture systems in cassava (*Manihot esculenta* Crantz). Nat. Biotechnol. 14, 726–730. 9630979 10.1038/nbt0696-726

[mpp12353-bib-0052] Taylor, N.J. , Masona, M.V. , Carcamo, R. , Ho, T. , Schöpke, C. and Fauquet, C.M. (2001) Production of embryogenic tissues and regeneration of transgenic plants in cassava (*Manihot esculenta* Crantz). Euphytica, 120, 25–34.

[mpp12353-bib-0053] Taylor, N.J. , Chavarriaga, P. , Raemakers, K. , Siritunga, D. and Zhang, P. (2004) Development and application of transgenic technologies in cassava. Plant Mol. Biol. 56, 671–688. 15630627 10.1007/s11103-004-4872-x

[mpp12353-bib-0054] Taylor, N. , Gaitan‐Solis, E. , Moll, T. , Trauterman, B. , Jones, T. , Pranjal, A. , Trembley, C. , Abernathy, V. , Corbin, D. and Fauquet, C.M. (2012) A high‐throughput platform for the production and analysis of transgenic cassava (*Manihot esculenta*) plants. Trop. Plant Biol. 5, 127–139.

[mpp12353-bib-0055] Tschuch, C. , Schulz, A. , Pscherer, A. , Werft, W. , Benner, A. , Hotz‐Wagenblatt, A. , Barrionuevo, L.S. , Lichter, P. and Mertens, D. (2008) Off‐target effects of siRNA specific for GFP. BMC Mol. Biol. 9, 60. 18577207 10.1186/1471-2199-9-60 PMC2443166

[mpp12353-bib-0056] Vanderschuren, H. , Moreno, I. , Anjanappa, R.B. , Zainuddin, I.M. and Gruissem, W. (2012) Exploiting the combination of natural and genetically engineered resistance to cassava mosaic and cassava brown streak viruses impacting cassava production in Africa. PLoS One, 7, e45277. 23049780 10.1371/journal.pone.0045277 PMC3458115

[mpp12353-bib-0057] Wagaba, H. , Beyene, G. , Trembley, C. , Alicai, T. , Fauquet, C. and Taylor, N. (2013) Efficient transmission of Cassava brown streak disease viral pathogens by chip bud grafting. BMC Res. Notes, 6, 516. 10.1186/1756-0500-6-516 PMC3896733 24314370

[mpp12353-bib-0058] Wang, H. , Beyene, G. , Zhai, J. , Feng, S. , Fahlgren, N. , Taylor, N.J. , Bart, R. , Carrington, J.C. , Jacobsen, S.E. and Ausin, I. (2015) CG gene body DNA methylation changes and evolution of duplicated genes in cassava. Proc. Natl. Acad. Sci. USA, 112, 13 729–13 734. 10.1073/pnas.1519067112 PMC4640745 26483493

[mpp12353-bib-0059] Winter, S. , Koerbler, M. , Stein, B. , Pietruszka, A. , Paape, M. and Butgereitt, A. (2010) Analysis of cassava brown streak viruses reveals the presence of distinct virus species causing cassava brown streak disease in East Africa. J. Gen. Virol. 91, 1365–1372. 20071490 10.1099/vir.0.014688-0

[mpp12353-bib-0060] Xu, P. , Zhang, Y. , Kang, L. , Roossinck, M.J. and Mysore, K.S. (2006) Computational estimation and experimental verification of off‐target silencing during posttranscriptional gene silencing in plants. *Plant* Physiol. 142, 429–440. 10.1104/pp.106.083295 PMC1586062 16920874

[mpp12353-bib-0061] Yadav, J.S. , Ogwok, E. , Wagaba, H. , Patil, B.L. , Bagewadi, B. , Alicai, T. , Gaitan‐Solis, E. , Taylor, N.J. and Fauquet, C.M. (2011) RNAi‐mediated resistance to Cassava brown streak Uganda virus in transgenic cassava. Mol. Plant Pathol. 12, 677–687. 21726367 10.1111/j.1364-3703.2010.00700.x PMC6640337

