# Supplementary figures and images for: Loss of CMD2‐mediated resistance to cassava mosaic disease in plants regenerated through somatic embryogenesis
Source: Mol Plant Pathol. 2016 Apr 5;17(7):1095–110. doi: 10.1111/mpp.12353 (PMC5021159; doi:10.1111/mpp.12353)

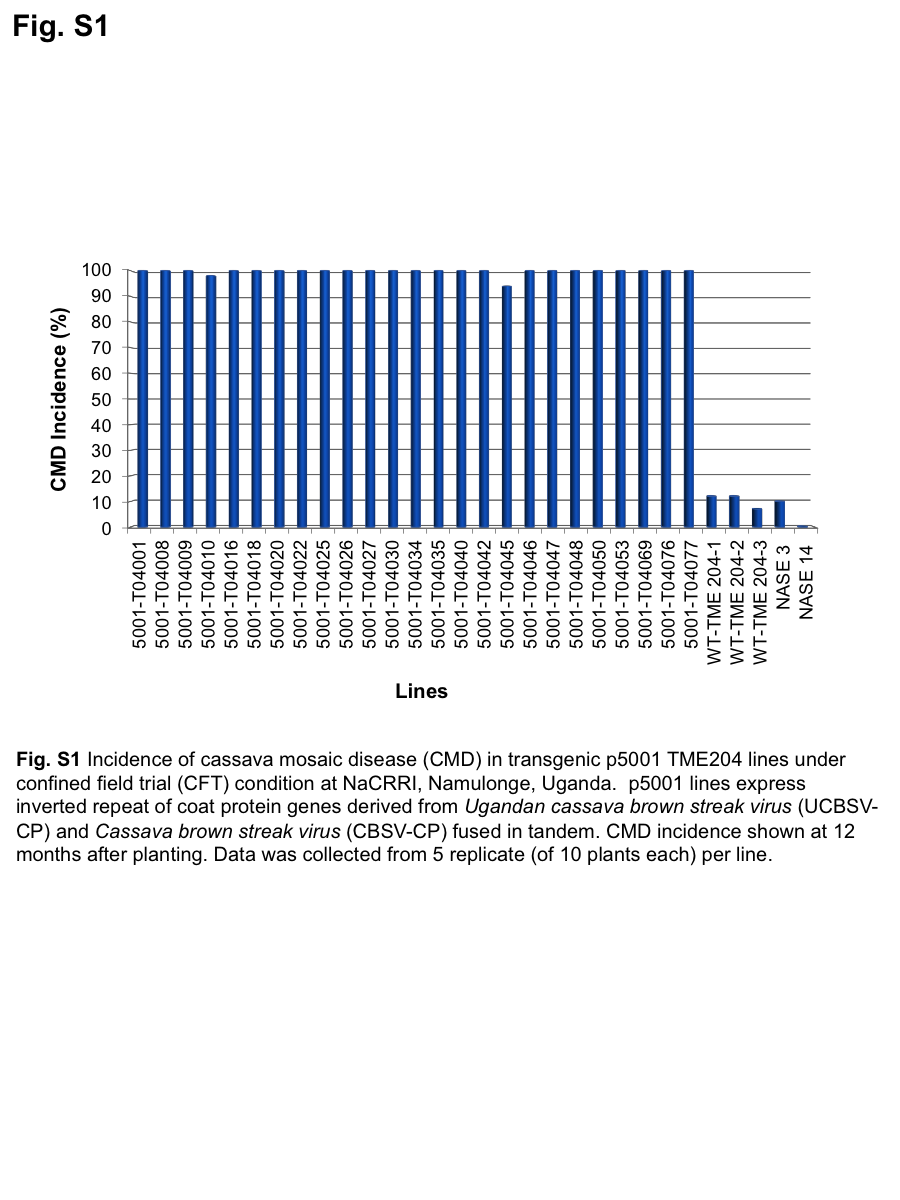

Supplement: Supplementary file 1 — Fig. S1 Incidence of cassava mosaic disease (CMD) in RNA interference (RNAi) p5001 TME 204 lines under confined field trial (CFT) conditions at NaCRRI, Namulonge, Uganda. p5001 lines express an inverted repeat of coat protein genes derived from Ugandan cassava brown streak virus and Cassava brown streak virus fused in tandem. CMD incidence shown at 12 months after planting. Data were collected from five replicates (10 plants each) per line. [file MPP-17-1095-s001.tiff]

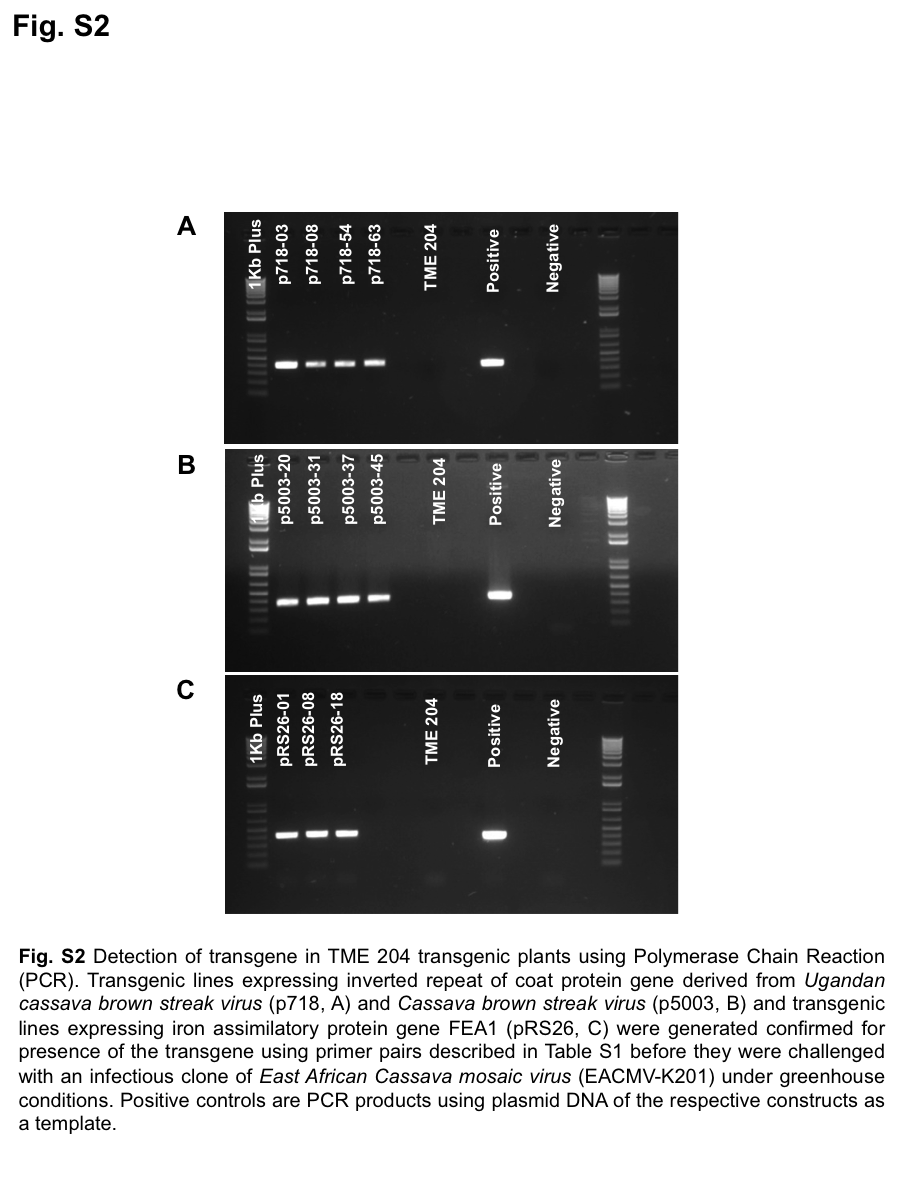

Supplement: Supplementary file 2 — Fig. S2 Detection of transgenes in TME 204 transgenic plants using polymerase chain reaction (PCR). Transgenic lines expressing the inverted repeat of the coat protein gene derived from Ugandan cassava brown streak virus (p718, A) and Cassava brown streak virus (p5003, B) and transgenic lines expressing the iron assimilatory protein gene FEA1 (pRS26, C) were generated and confirmed for the presence of the transgene using the primer pairs described in Table S1 before challenge with an infectious clone of East African cassava mosaic virus (EACMV‐K201) under glasshouse conditions. Positive controls are PCR products using plasmid DNA of the respective constructs as a template. [file MPP-17-1095-s002.tiff]

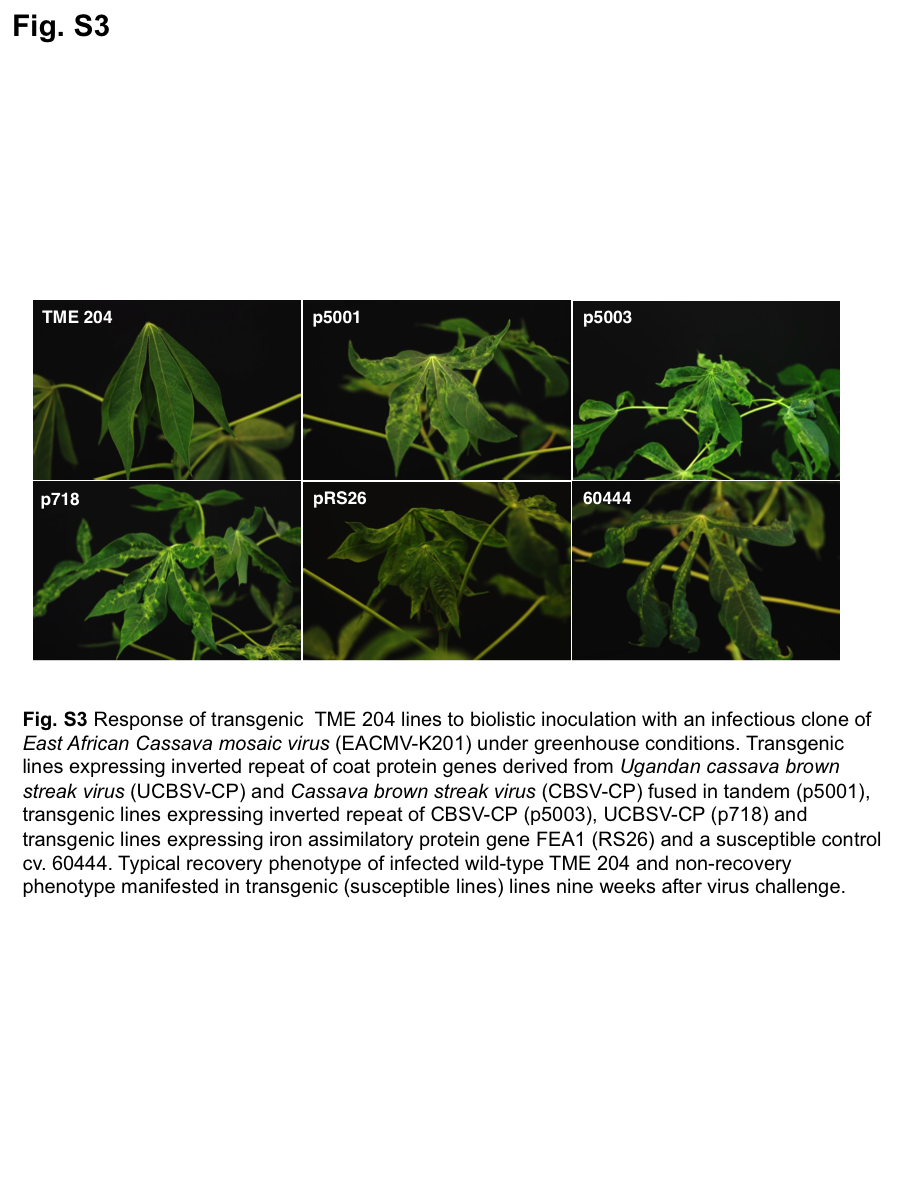

Supplement: Supplementary file 3 — Fig. S3 Response of transgenic TME 204 lines to biolistic inoculation with an infectious clone of East African cassava mosaic virus (EACMV‐K201) under glasshouse conditions. Transgenic lines expressing the inverted repeat of coat protein genes derived from Ugandan cassava brown streak virus (UCBSV‐CP) and Cassava brown streak virus (CBSV‐CP) fused in tandem (p5001), transgenic lines expressing the inverted repeat of CBSV‐CP (p5003) or UCBSV‐CP (p718), transgenic lines expressing the iron assimilatory protein gene FEA1 (pRS26) and susceptible control cv. 60444 were used. We observed the typical recovery phenotype in infected wild‐type TME 204 and non‐recovery phenotype in transgenic (susceptible lines) lines 9 weeks after virus challenge. [file MPP-17-1095-s003.tiff]

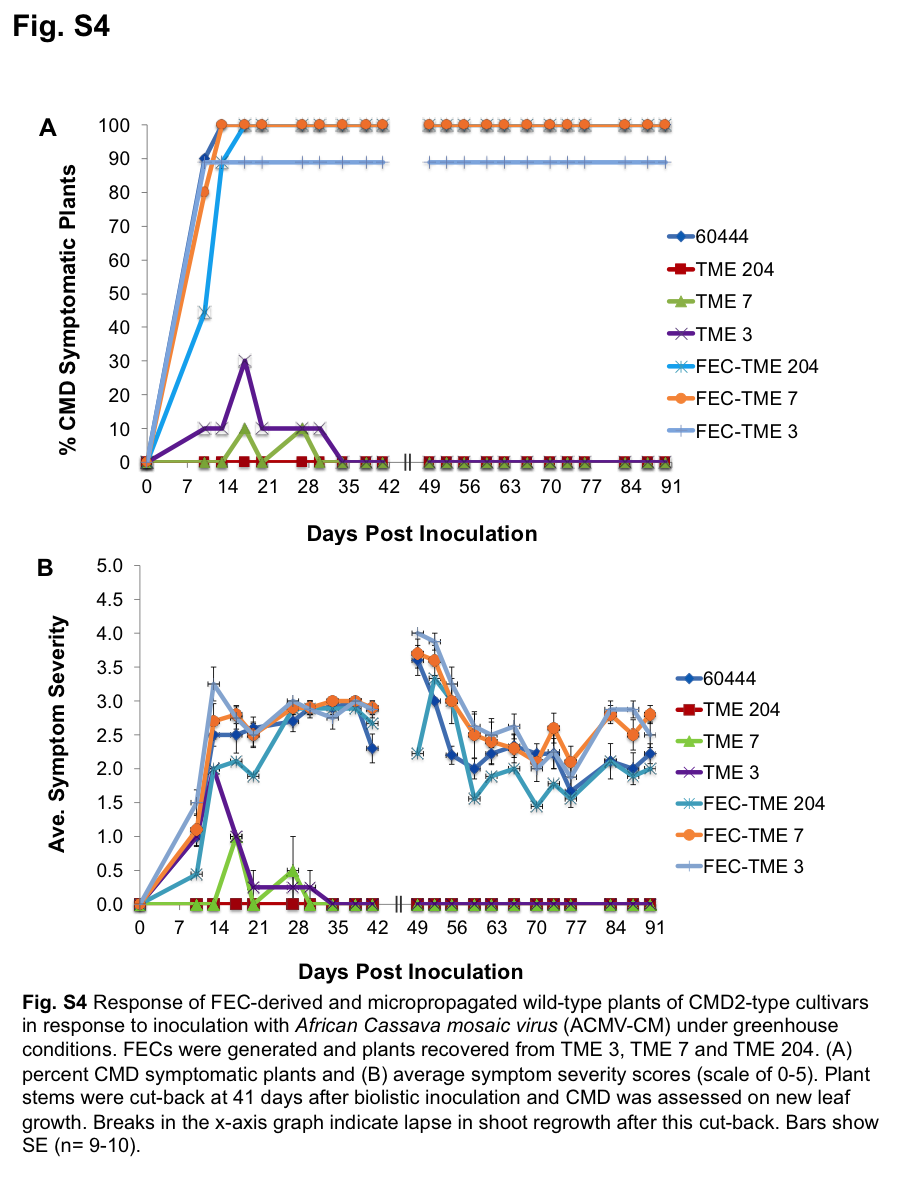

Supplement: Supplementary file 4 — Fig. S4 Response of friable embryogenic callus (FEC)‐derived and micropropagated wild‐type plants of CMD2‐type cultivars to inoculation with African cassava mosaic Cameroon virus (ACMV‐CM) under glasshouse conditions. FECs were generated and plants were recovered from TME 3, TME 7 and TME 204. (A) Percentage of cassava mosaic disease (CMD) symptomatic plants. (B) Average symptom severity scores (scale of 0–5). Plant stems were cut back at 41 days after biolistic inoculation and CMD was assessed on new leaf growth. Breaks in the x axis indicate a lapse in shoot regrowth after this cut back. Bars show standard error (n = 9–10). [file MPP-17-1095-s004.tif]

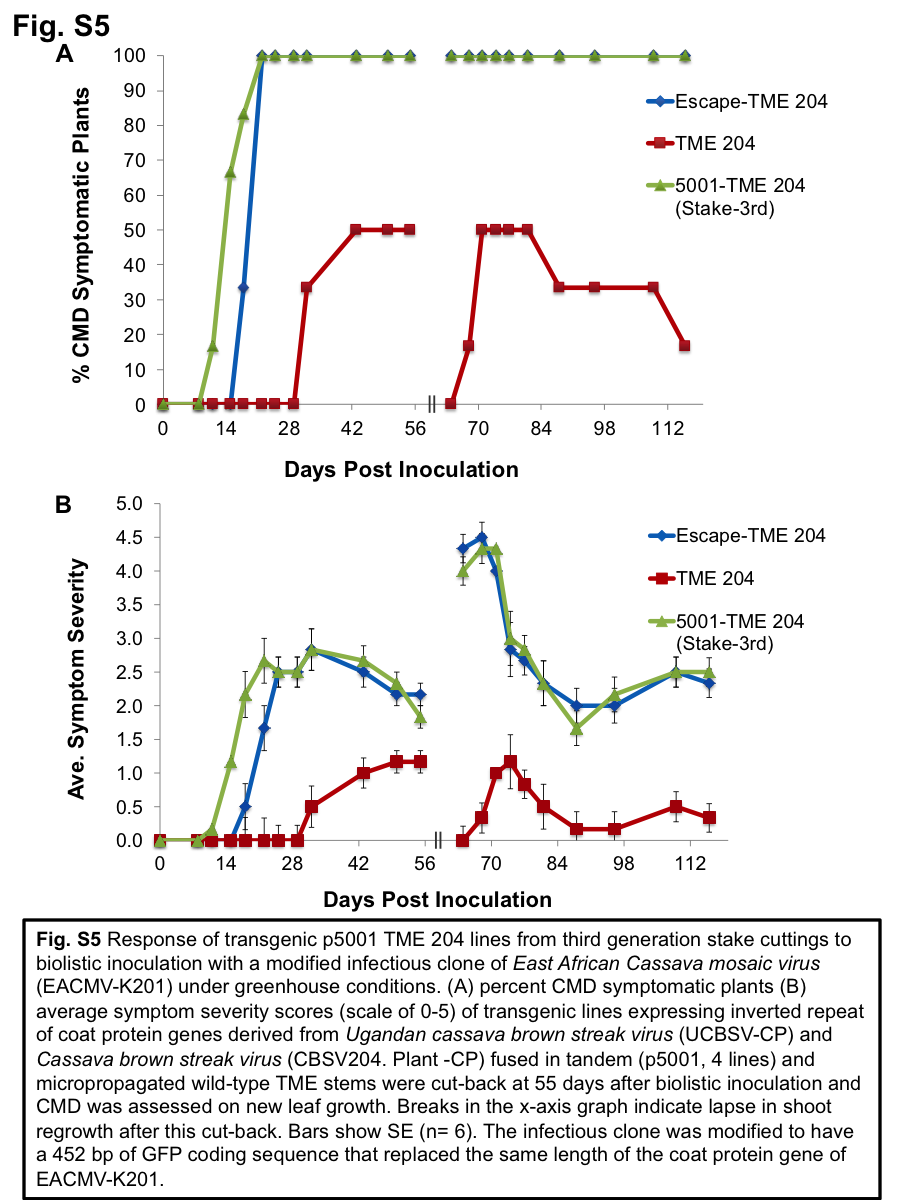

Supplement: Supplementary file 5 — Fig. S5 Response of transgenic p5001 TME 204 lines from third‐generation stake cuttings to biolistic inoculation with a modified infectious clone of East African cassava mosaic virus (EACMV‐K201) under glasshouse conditions. Percentage of cassava mosaic disease (CMD) symptomatic plants (A) and average symptom severity scores (scale of 0–5) (B) of transgenic lines expressing the inverted repeat of coat protein genes derived from Ugandan cassava brown streak virus and Cassava brown streak virus fused in tandem (p5001, four lines) and micropropagated wild‐type TME 204. Plant stems were cut back at 55 days after biolistic inoculation and CMD was assessed on new leaf growth. Breaks in the x axis indicate a lapse in shoot regrowth after this cut back. Bars show standard error (n = 6). The infectious clone was modified to contain 452 bp of green fluorescent protein (GFP) coding sequence that replaced the same length of the coat protein gene of EACMV‐K201. [file MPP-17-1095-s005.tif]
