# Supplementary material for: Loss of CMD2‐mediated resistance to cassava mosaic disease in plants regenerated through somatic embryogenesis
Source: Mol Plant Pathol. 2016 Apr 5;17(7):1095–110. doi: 10.1111/mpp.12353 (PMC5021159; doi:10.1111/mpp.12353)
Supplement: Supplementary file 6 — Table S1 List of primers used for transgene detection, labelling of probes and virus titre determination using quantitative polymerase chain reaction (qPCR). [file MPP-17-1095-s006.docx]

**Table S1**. List of primers used for transgene detection, labelling of probes and virus titre determination using quantitative polymerase chain reaction (qPCR)

| **Primer Name** | **Sequence** | **Target Gene (construct)** | **Product size (bp)** | **Purpose** | **Reference** |
| --- | --- | --- | --- | --- | --- |
| Pri227 | CCACCTTCTTGTAGCTCAGCTTGG | FEA1 (pRS26) | 409 | Transgene detection | This study |
| Pri228 | AACAACTGCGGCACCCTGTC |  |  |  |  |
| Pri069 | TGAAAGCCAGAATAATCAGCAA | p718 | 406 | Transgene detection | This study |
| Pri072 | CTGACATCGTGTGTCGTGTG |  |  |  |  |
| Pri13 | ATGGATACCACGTTCGCAAAGA | p5003 | 382 | Transgene detection | This study |
| Pri14 | GCACTGACGACAACAATGAAAAGA |  |  |  |  |
| Pri833 | TTGCAGAGGAAGATAGTGGGAATG | EACMV-K201 (DNA-A) | 306 | Probe for Sothern blot | This study |
| Pri834 | GAACGTGATGGGTTCCGCTG |  |  |  |  |
| Pri837 | TGCAAGGCTCACACTTTCATC | Serine/Threonine Protein Phosphatase (PP2A) | 227 | Reference gene for qPCR | Moreno et al. (2011) |
| Pri838 | CTGAGCGTAAAGCAGGGAAG |  |  |  |  |
| Pri1046 | GGTCTTCCCTGTACGACTATC | EACMV-K201 (DNA-A) | 108 | qPCR (virus load) | This study |
| Pri1047 | GGAACTTGAAGTCTGGGTTTCC |  |  |  |  |

Reference

Moreno, I., Gruissem, W., Vanderschuren H. (2011) Reference genes for reliable potyvirus quantitation in cassava and analysis of Cassava brown streak virus load in host varieties. J. Virol. Methods, **177**, 49**-**54.
